# Edible Herbal Medicines as an Alternative to Common Medication for Sleep Disorders: A Review Article

**DOI:** 10.2174/1570159X21666230621143944

**Published:** 2024-06-22

**Authors:** Azar Hosseini, Leila Mobasheri, Hassan Rakhshandeh, Vafa Baradaran Rahimi, Zohreh Najafi, Vahid Reza Askari

**Affiliations:** 1 Pharmacological Research Center of Medicinal Plants, Mashhad University of Medical Sciences, Mashhad Iran;; 2 Department of Cardiovascular Diseases, Faculty of Medicine, Mashhad University of Medical Sciences, Mashhad, Iran;; 3 Division of Biotechnology, Faculty of Veterinary Medicine, Ferdowsi University of Mashhad, Mashhad, Iran;; 4 International UNESCO Center for Health-Related Basic Sciences and Human Nutrition, Mashhad University of Medical Sciences, Mashhad, Iran;; 5 Applied Biomedical Research Center, Mashhad University of Medical Sciences, Mashhad, Iran

**Keywords:** Sleep, inflammation, herbal medicine, benzodiazepines, GABAergic system, oxidative stress

## Abstract

Insomnia is repeated difficulty in falling asleep, maintaining sleep, or experiencing low-quality sleep, resulting in some form of daytime disturbance. Sleeping disorders cause daytime fatigue, mental confusion, and over-sensitivity due to insufficient recovery from a sound sleep. There are some drugs, such as benzodiazepines and anti-histaminic agents, which help to sleep induction and insomnia cure. However, the prolonged administration is unsuitable because of tolerance and dependence. Therefore, the researchers attempt to find new medicines with lesser adverse effects. Natural products have always been good sources for developing new therapeutics for managing diseases such as cancer, cardiovascular disease, diabetes, insomnia, and liver and renal problems. Ample research has justified the acceptable reason and relevance of the use of these herbs in the treatment of insomnia. It is worth noting that in this study, we looked into various Persian herbs in a clinical trial and *in vivo* to treat insomnia, such as *Artemisia annua*, *Salvia reuterana*, *Viola tricolor*, *Passiflora incarnata*, lettuce, and *Capparis spinose*. According to research, herb extracts and fractions, particularly n-butanol fractions with non-polar agents, impact the benzodiazepine receptors and have hypnotic properties. Also, alkaloids, glycosides, flavonoids, saponins, and tannins in practically every plant are mentioned making them the popular natural compounds to help with sleep disorders and promote calmness.

## 1 INTRODUCTION

Insomnia is a widespread sleep disorder identified as sleep loss, insufficient sleep duration, or waking up multiple times during the night. It has been reported that 10 to 30 percent or even more of adults suffer from chronic insomnia [1]. Several factors can cause disturbance of these circadian rhythms and lead to neurological or non-neurological diseases. Studies have shown that sleep insufficiency has a role in the incidence of Alzheimer’s disease, depression, obesity, dyslipidemia, hypertension, and type 2 diabetes [2, 3]. There are chemical drugs such as benzodiazepine receptor agonists,histamine antagonists, and ramelteon (melatonin receptor agonists) that manage insomnia disorder [4, 5].

Despite their therapeutic effectiveness, these medications have various adverse effects when used. Headache and dizziness, psychomotor slowdown, memory and activity impairment, sadness and emotional lessening, anterograde amnesia, medication tolerance, and other adverse effects may be increased in the elderly [6, 7]. Therefore, studies have continued to find new hypnotic agents with lesser side effects and more efficacy. Herbal agents always have been a good source for developing new therapeutics for the treatment of some diseases, such as cancer [8, 9], immunodeficiency [10], cardiovascular [11], and abdominal aortic aneurism [12]. Herbal medicines are famous worldwide due to easy access, lower side effects, and cost-effectiveness. For many years, herbal medications have been utilized in folk medicine, and extracts of these medicines have been used to explore their pharmacological activities and mechanisms. Persian herbal remedies like *Nymphaea* spp., *Lactuca sativa, Crocus sativus*, and *Viola odorata* have been extensively recognized for improving sleep and other mental illnesses [13]. In this comprehensive review, we collected several Iranian medicinal plants that are often used to treat insomnia in animal or clinical studies (Tables **1** and **2**).

## 2 METHODS

In this review, documents were gathered that investigated the effects of Persian medicinal plants on sleep disorders, up to July 2022, from various databases, including Scopus, PubMed, Medline, and web of science. All relevant experimental and clinical studies were in English and included in the current review article (Tables **1** and **2**).

## 3 RESULTS

### 3.1 *Aloe vera*

*Aloe vera* (*A. vera*), with the name Sabre-zard, belongs to the Liliaceae family and is well-known in Persian traditional medicine [14]. It is known for its pharmacological effects, including moisturizing and anti-aging, digestive protection, wound healing, anti-inflammatory, laxative effect, anti-diabetic, anti-bacterial, anti-viral, anti-septic, improvement of convulsion, cerebral ischemia, and multiple sclerosis. The *A. vera* contains different compounds such as aloesin, barbaloin, emodin, acemannan, aloe-emodin, and polysaccharides [15, 16]. Also, Persian and international old pharmacopeias have reported sedative and hypnotic effects of *A. vera*. The development of an aqueous extract of *A. vera* leaves at doses of 50, 100, and 200 mg/kg on locomotion and pentobarbital-induced sleeping was investigated in rats (Table **1**). Administration of 200 mg/kg led to prolonged loss of righting reflex compared to the control group. Locomotion activity was repressed at doses of 100 and 200 mg/kg. Also, the extract and diazepam increased Non-rapid eye movement (NREM) sleep duration and decreased REM sleep [17]. The hypnotic effect of herbal medicine may be related to different herb compounds such as flavonoids and saponins [18]. Also, neurotransmitters such as acetylcholine and catecholamine are centrally acting anticholinergic, dopaminergic, noradrenergic, and serotonergic agents, causing a decrease in the duration and density of rapid eye movement (REM) sleep (Figs. (**1** and **2**) [17]. Studies have shown that *A. vera* increases acetylcholine *via* choline-esterase inhibition [19]. The presence of compounds with anti-acetylcholinesterase activity in *A. vera* can partly explain the observed changes in sleep impairment.

### 3.2 *Amygdalus communis*

*Amygdalus communis* (*Prunus amygdalus*, almonds) belongs to *Rosaceae* and grows in different regions of Iran. Other species could also be found in North Africa, the Balkan islands, Southwest Asia, Northeast Anatoly, Syria, Iraq, Lebanon, Afghanistan, Turkmenistan, and Central Asia [20]. *A. communis* is known for its pharmaceutical and nutritional importance. It is a rich source of triterpenoids, betulinic, ursolic, and oleanolic acids, phytosterols, as well as flavonol glycosides, and phenolic compounds [21].

**Fig. (1):**
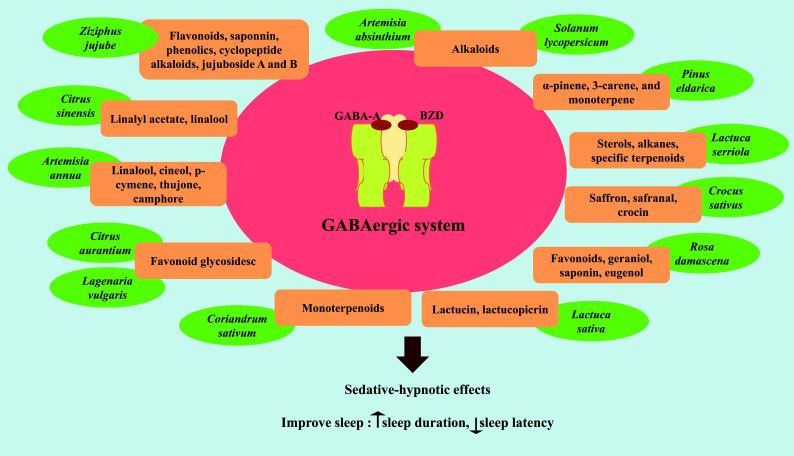
Herbal and its constituents-based treatments on the GABAergic system associated with sedative-hypnotic impacts. The GABAA receptor's primary isoform is α_1_β_2_γ_2_. The binding sites for the agonist GABA are situated at the β_2_^+^/α_1_^−^ subunit interfaces, whereas the modulatory site for benzodiazepines is located at the α_1_^+^/γ_2_^−^. Benzodiazepines are clinically relevant drugs that bind to GABA_A_ neurotransmitter receptors at the α_1_^+^/γ_2_^−^ interfaces, thereby enhancing GABA-induced chloride ion flux, leading to neuronal hyperpolarization. **Abbreviations:** GABA: gamma-aminobutyric acid, BZD: Benzodiazepine.

**Fig. (2):**
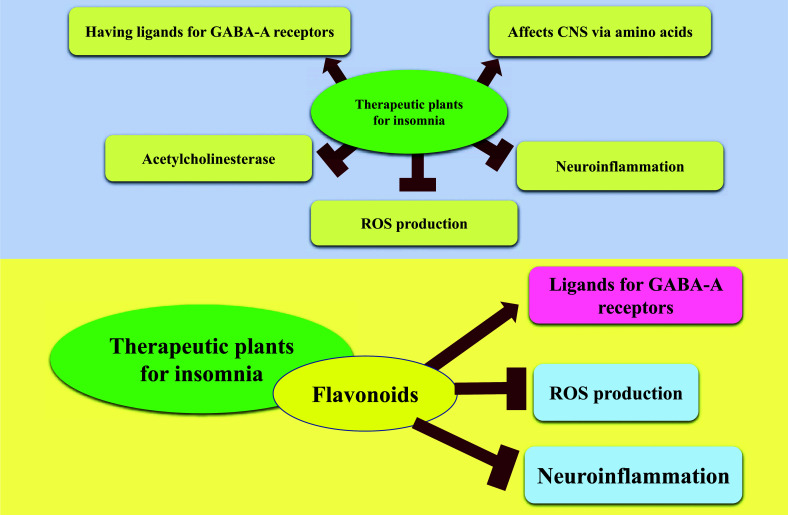
Therapeutic plants for insomnia act through different mechanisms.

It has been known by Iranian people since ancient times and has been used in Iranian foods due to its nutritional value. The oil from the seeds has also been used in skin and hair care products. Recent studies have suggested almonds' total and LDL cholesterol-lowering and HDL-increasing effects [22]. Current pharmacological studies represent that almonds have several biological activities, including prebiotic, antimicrobial, antioxidant, anti-inflammatory, anticancer, hepatoprotective, neuroprotection, anxiolytic, and sedative-hypnotic effects [23, 24]. The results of different studies in recent years have proven the importance of almonds in improving learning and memory and their positive impact on treating amnesia and Alzheimer's disease [25, 26]. The almond extract (100, 200, 400 mg/kg) was injected 30 min before pentobarbital administration (40 mg/kg). The rats were then gently positioned on their back every 15 s, and the onset of righting reflex loss was indicated, while no righting movements in response to repeatedly being positioned on its back were observed. Once the animal detected righting attempts, the time was recorded as the duration of pentobarbital-induced sleep or loss of righting reflex. This study showed that the extract at the 400 mg/kg dose increased sleep duration and suppressed locomotion activity. Also, the extract increased NREM and decreased REM (Table **1**) [27]. Certain amino acids in protein-rich plants are essential in their sedative/hypnotic properties. As an endogenous neurotransmitter, glycine affects the central nervous system (CNS) [28]. Serine and glycine have been associated with hypnotic effects (Figs. **1** and **2**). Also, glycine improves sleep quality in humans who complain of sleep disorders. Phenylalanine and tyrosine have shown positive effects on sleep patterns [29]. Almond has been proven to be rich in amino acids. This fact may underlie the almond extract's observed sedative/hypnotic effects. HPLC analysis identified amino acids such as glycine, phenylalanine, serine, and tyrosine as major constituents in the almond extract [30]. Therefore, the possible mechanism of the hypnotic effect of almonds may be related to their amino acids.

### 3.3 *Artemisia annua*

*Artemisia annua* is an aromatic annual herb that belongs to the *Asteraceae* family, endemic in the North of Iran [31]. Bioactive compounds, including flavonoids, terpenoids, coumarins, polyacetylenes, and sesquiterpenes (artemisinin), exert biological activities such as antimalarial, immunosuppressive, anti-inflammatory, and anti-tumorigenic activities [32, 33]. Also, it is composed of linalool, cineol, *p*-cymene, thujone, and camphor. These compounds have been studied to evaluate their effect on the CNS, presenting a facility to cross biological membranes because of their elevated liposolubility, which might affect the CNS [34]. In a study, the administration of *A. annua* essential oil (470 mg/kg) or crude ethanol extract (450 mg/kg) increased the immobility time and decreased activities such as ambulation, exploration, rearing, and grooming in animals (Table **1**). Furthermore, both essential oil and ethanol extract prolonged sleeping time and lost latency [34]. The main constituents of the *A. annua*, which present cineol, linalool, α-pinene, and p-cymene, might be responsible for their ability to enhance sleep [35, 36]. Chloroform, petroleum ether, and ethyl acetate fraction of *A. annua* with different concentrations (50, 100, and 200 mg/kg) were administered intraperitoneally (i.p.) in male mice (Table **1**). Flumazenil (3 mg/kg, i.p.) as a benzodiazepine (BZD) receptor antagonist was injected 15 min before extract. Chloroform fraction significantly increased immobility time in a dose-dependent manner. In contrast, flumazenil decreased immobility time induced by chloroform fraction significantly. This study showed that *A. annua* has sedative effects, which are probably mediated *via* BZD receptor pathways [37]. According to two studies, the sedative effect of *A. annua* is related to active compounds and BZD receptors.

### 3.4 *Citrus aurantium*

*Citrus aurantium*, commonly known as sour orange or bitter orange (local name in Iran: Nareng), is produced in Northern and Southern Iran [38]. Persian traditional medicine considers the *Citrus* genus to be beneficial in reducing anxiety or sleeplessness symptoms, and *C. aurantium* has lately been recommended as an antidepressant adjuvant [39].

Carvalho-Freitas and Costa showed that the essential oil from *Citrus aurantium L.* peel (1.0 g/kg) increased the sleeping time induced by barbiturates, and the time spent in open arms significantly is indicative of sedative and anxiolytic effects (Table **1**). It also caused an anticonvulsant effect [40]. Motaghi *et al.* evaluated the anxiety and sedative effects of *Citrus aurantium* L. flowers in rats. The treated groups received 62.5, 125, and 250 mg/kg (i.p.) of aqueous extract, and after 30 min, each animal was administered sodium pentobarbital. Administration of 62.5 and 125 mg/kg of aqueous extract of *C. aurantium* L. flowers caused a significant increase in the percentage of time spent in the open arms, a substantial decrease in closed arms, and reduced locomotor activity. Furthermore, *C. aurantium* L. aqueous extract at 125 mg/kg and 250 mg/kg significantly prolonged the duration of pentobarbital sleeping time and shortened the onset of sleep in rats [41]. Carvalho-Freitas *et al.* showed that peeling essential oil, Hexane, and dichloromethane fractions (1.0 g/kg) of *Citrus aurantium* enhances the sleeping time induced by barbiturates (Table **1**) [40]. In a clinical trial, aromatherapy with *Citrus aurantium* significantly influenced the time it takes to fall asleep, the duration of sleep, and the ability to go asleep again after being up for a length of time in cardiovascular patients (Table **2**) [42]. *C. aurantium* L. mechanism on the CNS can be related to flavonoid glycosides (naringin, hesperidin, and neohesperidin, flavones, flavonones, polimethoxylates, glycan peptides) existing in it. Many flavonoids were found to be ligands for the γ-amino butyric acid type A (GABA-A) receptors in the CNS, which led to the hypothesis that they act as benzodiazepine-like molecules (Figs. **1** and **2**) [42, 43]. In addition, Fernandez *et al.* detected the anxiolytic and sedative effects of a range of flavonoid glycosides (myrcitrin, naringin, and gossypin) in mice [44].

### 3.5 *Coriandrum sativum*

*Coriandrum sativum* (*C. sativum*) is an annual herb belonging to the *Apiaceae* family, which is extensively used as a spice, as well as in the pharmaceutical and food industries. It is also known as coriander, cilantro, Chinese parsley, and “*Geshniz*” in Persian. Although all parts of the plant are edible, its fresh leaves and dried seeds are most frequently used in many cultures [45]. In traditional medicine, *C. sativum* is commonly used to treat nervousness, vertigo, headache, swelling, fever, digestive problems, respiratory infections, allergies, and wounds [46]. The main bioactive constituents in *C. sativum* are essential oil (1-ethenyl-cyclododecanol, (E)-2-Decenal, phytol, linalool, citronellol, dodecenal), fatty acids (petroselinic acid, linoleic acid, palmitic acid, and oleic acid), tocopherol and tocotrienol, sterol (β-sitosterol, stigmasterol), carotenoids (β-carotene, lycopene lutein, zeaxanthin) and polyphenols [47]. Experimentally, *C. sativum* has reported to have a wide range of biological activities, including anti-inflammatory, antidiabetic, hypolipidemic, neuroprotective, hepatoprotective, analgesic, antioxidant, and anticonvulsant effects [48-50]. Some research suggest that *Coriandrum sativum* extract has sedative and anxiolytic properties. In a study, i.p administration of the aqueous extract (200, 400, and 600 mg/kg), hydro-alcoholic extract (400 and 600 mg/kg), and essential oil (600 mg/kg) of *Coriandrum sativum* L. seeds prolonged pentobarbital-induced sleeping time in mice (Table **1**) [51]. In another study, *C. sativum* seed extract administered orally (250, 500, and 1000 mg/kg) to mice failed to exert effects on sleep onset and duration (Table **1**) [52]. Gastَn *et al.* indicated that intracerebroventricular injection of essential oil from *Coriandrum sativum* seeds (8.6 and 86 μg) induced a sedative effect in neonatal chicks [53].

The action of the plant might be attributed to linalool, the primary coriander component, which has various neuropharmacological effects such as anti-anxiety, sedative, and anticonvulsant [54]. Other monoterpenoids such as limonene, myrcene, γ-terpinene, and α-pinene in *C. sativum* are shown to possess sedative and anxiolytic effects due to the interaction of monoterpenes with γ-Aminobutyric acid type A (GABAA) receptors [55, 56]. GABAA receptors are key inhibitory neurotransmitter receptors in a variety of neuropsychiatric disorders. GABAA receptors can be activated and regulated by a variety of medicines. Diazepam and other benzodiazepines are well-known medications that operate as positive allosteric modulators of a subset of these receptors. They are sedative, anxiolytic, anticonvulsant, hypnotic, and have anticonvulsant and muscle relaxant characteristics. The GABAA receptor's primary isoform is α_1_β_2_γ_2_. The binding sites for the agonist GABA are situated at the β_2_^+^/α_1_^−^ subunit interfaces, whereas the modulatory site for benzodiazepines is located at the α_1_^+^/γ_2_^−^ (Fig. **1**) [57, 58]. According to Sakurai *et al.*, the sedative action of coriander leaf extract is attributable to the hyperactivity of inhibitory neurons in the brain because the leaf extract (600 mg/kg) raised the gene expression of the GABA-A receptor-1 subunit and decreased the gene expression of GABA transporter (Table **1** and Fig. **2**) [59].

### 3.6 *Lactuca sativa*

*Lactuca sativa* (lettuce) belongs to the *Compositae* family and is a farmed and highly consumed vegetable worldwide. Although lettuce is a popular vegetable, it has not been considered a nutritional food owing to its high water content. However, depending on the variety of lettuce, the nutritional content might equal that of other “nutritious” plants [60].

Different types of lettuce contain different amounts of compounds, including dietary fiber, vitamins (vitamins A, C, K, folate, niacin, riboflavin, thiamine), phenolic compounds, chlorophyll, b-carotene, lutein content, minerals (N, P, Mg, Zn, Ca, Fe, K, Mn, Se), isorhamnetin, quercetin, kaempferol, epicatechin, myricetin, anthocyanin, saponins. Alkaloids, tannins, and steroids are associated with anti-oxidant, radical scavenging, anti-inflammatory, anti-cancer, anti-cataracts, and anti-cardiovascular disease activities [61-63]. Traditionally, lettuce has been suggested to have sedative-hypnotic properties. The hydro-alcoholic extract of *Lactuca sativa (*400 mg/Kg*) and its* n-butanol fraction *(*200 mg/Kg*)* prolonged the pentobarbital-induced sleep duration and decreased sleep latency in rats that may be exerted by the non-polar agents (sterols, alkanes, and some terpenoids) in an n-butanol fraction of this plant (Table **1**) [64]. *Lactuca sativa* seed extract contains caftaric acid, chlorogenic acid, and chicoric acid, significant antioxidant phenolics that protect against oxidative stress produced by sleep disruption [65]. Kim *et al.* demonstrated that orally administering 100 mg/kg of green romaine leaf extract facilitates the effect of pentobarbital-inducing sleep by decreasing latency, extending sleep duration, and improving sleep quality by boosting NREM. These findings indicate that lactucin and lactucopicrin, which are found in green romaine leaf extract, bind efficiently to GABAA receptors and serve as the active chemical that induces sleep [66]. In a clinical trial study, the administration of capsules containing lettuce seed (1000 mg) improved the quality of sleep in pregnant women with sleeplessness for 14 days (Table **2**) [67]. In another study, patients with breast cancer suffering from sleep disorders were treated with lettuce seed syrup (5 mL), and their insomnia symptoms improved (Table **2**) [68].

### 3.7 *Lactuca serriola*

*Lactuca serriola* L. (Compositae) is an annual or biennial plant known by several names, including Prickly lettuce, Compass weed, Jagged lettuce, Kahu, and Khas. It is native to the Himalayas, Siberia, and Atlantic areas but is also cultivated in temperate lands of Europe, Asia, and Africa [69]. The phytochemical screening of the *Lactuca serriola* showed the presence of alkaloids, glycosides, carotene, carbohydrates, triterpenoids, tannins, saponins, phytosterols, phenolic compounds, flavonoids, triterpenoids, vitamins (B1, C, E, K), minerals (Na, K, Mg, S, Cl, P), organic acid (oxalic acid), and sesquiterpene esters in seeds, leaves, and stems of the plant [70]. In addition, they have antimicrobial, antioxidant, anti-venom, anticancer, antispasmodic, diuretic, anesthetic, bronchodilatory, vasorelaxant, demulcent, and hepatoprotective properties [71, 72]. *Lactuca serriola* is sometimes known as wild opium because its latex contains analgesic and sedative compounds [73].

The hydro-alcoholic extract of *Lactuca serriola* aerial parts (50-400 mg/kg) increased sleeping time and lowered latency to fall asleep, comparable to diazepam, which may operate *via* benzodiazepine receptors. Also, the n-butanol fraction (400 mg/kg) caused a sedative effect among the other fractions but not more than the hydro-alcoholic extract, suggesting that the active molecules responsible for the impact of lettuce are non-polar agents such as sterols, alkanes, and specific terpenoids (Table **1**) [74]. According to studies, Terpenoids with diverse chemical structures exhibit varying affinities for the GABA receptor and modify GABA receptors either by contact with a common BZD site on the receptor or independently of BZD sites (Table **1**) [75, 76].

### 3.8 *Lavandula angustifolia*

*Lavandula angustifolia Mill.* (*also known as Lavandula officinalis Chaix*), lavender belongs to the Lamiaceae family, which is mainly native to the Mediterranean region, although it is widely grown in England, Europe, North America, and Australia [*77*]. The major constituents of *lavender* essential oil are 1,8-cineole, camphor, camphene, *α*-pinene, *β*-pinene, p-cymene, limonene, terpinen-4-ol, cryptone, T-cadinol, borneol, 3-carene, linalool, lavandulyl acetate, linalyl acetate [78, 79]. Lavender has a long history of medicinal use, and according to animal and clinical studies, it is used as herbal medicine to relieve stress, anxiety, and neuropathic pain, most likely due to an NMDA-receptor antagonism, inhibition of the serotonin transporter and decreased levels of iNOS in the spinal cord [80, 81]. Lavender has also been used to promote sleep. Alnamer *et al.* demonstrated that methanolic (200, 400, and 600 mg/kg) and aqueous (200 and 400 mg/kg) extracts of *Lavandula officinalis* L. had sedative and hypnotic effects in mice when compared to diazepam, which was mediated through the GABAergic system (Table **1**) [82]. For four weeks, lavender aromatherapy improved sleep quality, quantity, mood, and life quality in diabetic patients with sleeplessness (Table **2**) [83]. A randomized pilot study showed aromatherapy of *L. augustifolia* improved sleep in patients with mild insomnia (Table **2**) [84]. A clinical trial was performed on 64 patients with ischemic heart disease. The intervention included three nights, each time 9 hours of aromatherapy with lavender oil for the experiment group. The sleep quality in ischemic heart disease patients improved after aromatherapy with lavender oil (Table **2**) [85].

### 3.9 *Leonurus cardiaca*

*Leonurus cardiaca* L. (Motherwort) is a perennial herb of the Lamiaceae family, initially in Asia and Southeastern Europe but is now found worldwide [86]. Some components from various chemical categories have been found in the *Leonuri cardiacae*, including lavandulifolioside, stachydrine, stereoisomers, ursolic acid, chlorogenic acid, leocardin, leonurine, galiiridioside, reptoside, alkaloids and choline with antimicrobial, antioxidant, anti-inflammatory, analgesic, uterotonic, cardiovascular, neuroprotective and sedative actions [86, 87]. In a clinical study, Leonurus oil extract (1200 mg/day) showed sedative effects and improvement in the symptoms of anxiety and sleep disorders in patients with arterial hypertension [88]. Furthermore, it has been demonstrated that the motherwort extracts with glycine, valine, and arginine reduced anxiety in animals [89]. According to research, the neurological mechanism of action of *Leonurus cardiaca* primarily depends on its interaction with the GABA site of the GABA type A receptor (Table **1**) [90].

### 3.10 *Nepeta glomerulosa*

*Nepeta glomerulosa* (Lamiaceae family) is one of the Nepeta genus species, consisting of about 300 species widely distributed in Europe, Asia, and some areas of Africa. Seventy-nine species of Nepeta, with the common Persian name of “Pune-sa”, are distributed in Iran (particularly in the provinces of Khorasan and Isfahan) [91]. The significant components of *Nepeta glomerulosa* oil are monoterpenes, oxygenated monoterpenes, 1,8-cineole, stapfiana, and caryophyllene oxide [92, 93]. It is widely used in the folk medicine of Iran for digestive disorders, antimicrobial, eye illnesses, respiratory disorders, diuretic, diaphoretic, febrifuge, and sedative effects [91, 94].

Hosseini *et al.* showed that the hydro-alcoholic extract (50-200 mg/Kg) and an n-butanol fraction (50 and 100 mg/Kg) increased sleep duration and decreased sleep latency *via* the GABAergic system in the pentobarbital-induced sleep model without any cytotoxicity (Table **1**). The components responsible for this effect are most likely non-polar agents found in the n-butanol fraction. Therefore, the non-polar agents identified in the n-butanol fraction are most likely responsible for this impact [95].

### 3.11 *Citrus sinensis*

*Citrus sinensis* L., sweet orange, is a small tree in the Rutaceae (citrus) family that originated in Asia and spread over the tropical regions of the world [96]. Flavonoids, steroids (-sitosterol), linalyl acetate, linalool, hydroxyl-amides, alkaloids (synephrine and octopamine), protoalkaloids, coumarins, carbamates, carotenoids, triterpenes, vitamin C, and pectin have all been discovered in the peel, leaves, flowers, and oil of *C. sinensis.* [96, 97]. *C. sinensis* peel, as a natural radical defense, has an essential role in various disorders, including cancer, cardiovascular dysfunction, neurological diseases, gastrointestinal disease, inflammation, and aging [98]. In addition, *C. sinensis* is traditionally used as a sedative, hypnotic, and anxiolytic [96, 99].

In a triple-blind randomized controlled clinical trial, the orange peel essential oil positively improves mothers’ sleep quality in the postpartum period. Orange peel essential oil has active ingredients such as linalyl acetate and linalool that have been reported to have narcotic effects through interaction with the GABA (gamma-aminobutyric acid) receptor [100]. Also, hesperidin, the main flavonoid in *C. sinensis*, was identified as the active principle in this plant responsible for sedation (Table **2**) [101].

### 3.12 *Pinus eldarica*

*Pinus eldarica* (*Pinaceae*) is an evergreen tree native to the Transcaucasia region between Europe and Asia. It is one of Iran's most common pines and grows in Afghanistan and Pakistan [102]. *P. eldarica* contained high amounts of polyphenolic compounds such as catechin, tyrosol, epicatechin, gallic acid, vanillic acid, ferulic acid, and coumaric acid [103]. *P. eldarica* oil was primarily composed of mono- and sesquiterpenoid fractions, particularly α-pinene, caryophyllene oxide, δ-3-carene, (E)-β-caryophyllene, and myrtenal [104]. In Persian traditional medicine, it was reported that plants of the Pinaceae family have sedative and hypnotic effects. A study showed that the hydro-alcoholic extract (25-200 mg/kg) and an n-butanol fraction (25 and 50 mg/kg) of *P. eldarica* decreased sleep latency and significantly increased the duration of sleep induced by pentobarbital. As a result, it can be inferred that the active ingredients of *P. eldarica* responsible for sleep prolongation include low polar agents in an n-butanol fraction, such as alkanes, sterols, and terpenoids [105]. Also, studies showed that α-pinene, and 3-carene, monoterpene of the family *Pinaceae*, improved sleep quality through binding to the BZD site of α_1_ and g_2_ subunits of GABA_A_-BZD receptor (Table **1**, Fig. **1**) [106, 107].

### 3.13 *Stachys lavandulifolia*

*Stachys lavandulifolia* Vahl (*Lamiaceae*), one of the species of the genus Stachys, is an aromatic plant that grows in different regions of Iran, including Azerbaijan, Golestan, Khorasan, Behshahr, Mazandaran, and Tehran provinces [108]. The primary ingredients of the essential oils of *S. lavandulifolia* are 4-hydroxy-4-methyl-2-pentanone. α-thujone, α-pinene, myrcene, β-phellandrene, germacrene D, Δ-cadinene, 1, 4-methano-1-H-indene, hexadecanoic acid, lavandulifolioside-B, and 5-*O*-β-allopyranosyloxy-aucubin [108-110]. The traditional usage of *S. lavandulifolia* for its hypnotic and sedative properties has been significant [111]. Rabbani *et al.* showed that intraperitoneal doses of 100 and 300 mg/kg of *S. lavandulifolia* extract significantly prolonged sleep duration and diminished the locomotor activity in treated mice, which probably is mediated by volatile oil and phenyl propanoid glycoside (Table **1**) [112].

### 3.14 *Salvia leriifolia*

*Salvia leriifolia (*Lamiaceae*)*, also known as Noruzak and Jobleh, is a perennial herbaceous plant that grows exclusively in Khorasan and Semnan provinces, Iran [113]. Chemical composition of the essential oil of *S. leriifolia*, including α- and β-pinene, camphene, Δ-3-carene, ρ-cymene, 1,8-cineole, borneol, terpinen-4-ol, α-terpineol, α-muurolene, y-cadinene, Δ-cadinene, 10-epi-gamma-eudesmol, α-cadinol [114]. In recent years, this plant has been studied for the benefits of antidiabetic, pain relief, anti-inflammatory, antioxidant, antiulcer, antibacterial activity, anti-carcinogenic, and sedation [113]. Hosseinzadeh and Hassan-Zadeh demonstrated that an aqueous extract of *S. leriifolia* extended sleeping duration and promoted muscular relaxation in mice [115]. Pretreatment of animals with compounds from ethanol extract of the *S. leriifolia* (10, 15, and 20 mg/kg) caused a significant seductive and muscle relaxant-like effect through interaction with GABAA receptor similar to that of BDZ (Table **1**) [116].

### 3.15 *Salvia reuterana*

*Salvia reuterana* Bioss. is one of the 61 species of the genus *Salvia* L. in the *Lamiaceae* family. In Persian, this scented perennial plant is known as “Maryam Goli-e Esfahani.” [117]. The major components of the *S. reuterana* oil are germacrene D, b-caryophyllene, bicyclogermacrene, caryophyllene oxide, and spathulenol [118]. *S. reuterana* can be utilized to treat various diseases, including cancer, diabetes, microbiological, oxidative, and neurological illnesses [119]. It has been used in Persian traditional medicine for sedative and anxiolytic effects. Vaseghi *et al.* discovered that an ethanolic extract of *S. reuterana* (50, 100, and 250 mg/kg) decreased latency and increased total sleeping time in ketamine-induced sleeping mice [120]. The hydro-alcoholic extract of *S. reuterana* Boiss. (100 mg/kg) possess anxiolytic and sedative effects in mice (Table **1**) [121]. *S. reuterana* components likely induced sedation by inhibiting acetylcholinesterase or contacting GABA receptors [111].

### 3.16 *Valeriana officinalis*

*Valeriana officinalis* L. or Valerian (Caprifoliaceae) is a medicinal herb native to Europe, Asia, and North America. Various parts of the plant are used to treat stomach issues, neuronal disorders, and urinary tract infections [122]. Valerian essential oils are reported for their sedative and anxiolytic activity, suggesting that their compounds act synergistically [123]. Valerian components, including sesquiterpenes (valerinic acid, valeranone), triterpenes (ursolic acid), monoterpenes (borneol, bornyl acetate), valepotriates (valtrate, didrovaltrate, isovalerenic acid), flavonoids, lignans, alkaloids (valerine) have been shown in several experiments to have biological action [124]. A pilot study investigates a combination of valerian and hop (Ze 91019) in 30 patients suffering from mild-moderate, non-organic insomnia. The patients were treated with two tablets (250 mg valerian extract and 60 mg hop extract) in the evening. They reported an improvement in sleep after two weeks of treatment (Table **2**) [125]. Also, the valerian/lemon balm used in 100 women aged 50-60 who complained of sleep disorders reduces symptoms of sleep disorders during menopause [126]. In addition, a study was conducted on 90 patients with acute coronary syndrome (ACS) at Mazandaran Heart Center, Mazandaran, Iran. Patients in the acupressure with valerian oil 2.5% group (*i.e*., valerian acupressure group) received bilateral acupoint massage with two drops of valerian oil for 2 minutes for three nights. Results showed that using these techniques can significantly improve sleep and reduce waking during the night (Table **2**) [127]. Valerian is known to stimulate the release of neurotransmitters such as GABA and inhibit the enzyme-induced breakdown of GABA in the brain, perhaps acting as a precursor for GABA synthesis. However, it has been discovered that valerian lignan hydroxy pinoresinol binds to benzodiazepine receptors [123, 128].

### 3.17 *Viola tricolor*

*Viola tricolor* L. (Heartsease), a Violaceae plant family member, is a popular gardening plant in Iran [129]. The essential oil obtained from aerial parts of *V. tricolor* has compounds including sesquiterpenes, monoterpenes, flavonoids, shikimic acid, aliphatics, bisabolone oxide, trans-β-farnesene, hexahydrofarnesyl acetone, methyl salicylate, and β-ionone [130]. Studies showed various therapeutic properties of *V. tricolor,* including anti-angiogenesis, anti-apoptotic and anti-proliferation of cancer cells, anti-inflammatory, immunosuppressive activity, antimicrobial, and neuronal cell protection [129, 131-136]. Traditionally, *V. tricolor* has been suggested to have sedative-hypnotic properties. However, Ghorbani *et al.* revealed the hydro-alcoholic extract of *V. tricolor* at 300 mg/kg, significantly prolonged the duration of pentobarbital-induced sleep with no neuron toxicity (Table **1**). Similarly, the ethyl acetate fraction significantly augmented the sleep length, and none of them could dramatically change the sleep latency time [137]. Furthermore, some of the chemicals in *V. tricolor*, such as rutin, have been shown in studies to alleviate anxiety by activating the GABAergic system in the basolateral amygdala and may also enhance the GABAergic systems (Table **1**, Figs. **1** and **2**) [138].

### 3.18 *Viola odorata*

*Viola odorata* L. (Sweet Violet), a member of the *Violaceae*, is known as Banafshe in Farsi and is found in Northern Iran, particularly in the Alamut region [139, 140]. In current phytotherapy, these herb plants possess antibacterial, anti-inflammatory, antioxidant, antipyretic, sedative, neuropharmacological, vasculoprotective, and hepatoprotective activities [140-143].

In a clinical investigation, researchers discovered that using 66 mg of *V. odorata* intranasal in each nostril before bed for one month improved sleep in individuals with chronic insomnia (Table **2**) [144]. Also, in another study, the administration of *V. odorata* oil nasal drop can alter sleep start, degree of hypnotic medication, sleep quality indices, mental quality of sleep, and sleep duration in older persons [145]. Shayesteh *et al.* documented that administering 5 mL *V. odorata* syrup for four weeks increases the sleep quality index in depression and obsessive-compulsive disorder patients [146]. Monadi *et al.* observed in rats that an injection of 400 mg/kg of *V. odorata* extract caused an increase in sleeping time as well as sedative and anxiolytic effects superior to diazepam (Table **1**) [147].

Numerous studies have proven that the essential oil of *V. odorata* flowers is rich in polyphenols, monoterpenes, sesquiterpenes, linalool, and other antioxidant and neuroprotective characteristics that might explain its hypnotic effects [144, 148]. Furthermore, melatonin, which has been extensively studied as a dietary supplement for its hypnotic features, is found in *V. odorata* flowers [149].

### 3.19 *Ocimum basilicum*

*Ocimum basilicum* L. (Basil) is a well-known aromatic *annual or perennial plant in the genus Ocimum (basil) and family Lamiaceae, native to Africa, India, and Asia and widely grown in temperate climates across the world* [150]. Monoterpenes, sesquimonoterpenes, triterpenes, aromatic substances, aliphatic compounds, flavonoids, monosaccharides, coumarin, cinnamates, polyphenols, glycosides, steroids, and miscellaneous compounds make up the chemical composition of its essential oils [150].

It has been reported to have antimicrobial, anti-tumor, antispasmodic, aromatic, carminative, anti-dyspepsia, antihyperlipidemic, snake bites and skin problems, anti-giardia, antiinflammatory, febrifuge analgesic, antioxidant, antiulcer, blood-sugar-lowering, insecticidal, anti-aging, wound-healing, sedative and platelet aggregation inhibiting properties [151, 152]. Also, it affects the central nervous system (CNS) and treats several neurological conditions. Several experiments were conducted to examine the antidepressant efficacy of *O. basilicum* L. extract. Researchers discovered that the methanolic extract of *O. basilicum*, due to its antioxidative potency and free radical scavenging activity, attenuates the depressant-like actions against oxidative damage in rats [153].

Askari *et al.* discovered that hydro-alcoholic extract of *O. basilicum* (25, 50, or 100 mg/kg), ethyl acetate (50mg/kg), n-butanol (50 mg/kg), and aqueous fractions (50 mg/kg) increased sleep duration and, while sleep latency was significant in hydro-alcoholic and n-butanol fractions (Table **1**) [154]. The possible mechanism for sedative-hypnotic effects of *O. basilicum* could be associated ‎with‎ the‎ presence ‎of‎ non-polar agents such as linalool, eugenol, bergamotene, germacrene D, cadinene, cadinene, selinene, and spathulenol identified in the extracts of *O. basilicum* [155].

### 3.20 *Artemisia absinthium*

*Artemisia absinthium* (Asteraceae), sometimes known as Wormwood or Afsantin, is a medicinal herb used in Europe, West Asia, North America, and Australia. The following substances are primarily responsible for the herb's biological activity: the essential oil (thujyl alcohol esters, octane, α-pinene, linalool, *etc*.), bitter compounds (absintholide, absinthin, anabsinthin, artabin, artabsin, artamarin, azulene), flavonoids (quercetin, naringenin, artemetin, rutoside), other bitterness-imparting compounds, phenolic acids (chlorogenic acid, ferulic acid, gallic acid, caffeic acid, coumaric acid, salicylic acid, rosmarinic acid, tannic acid, syringic acid, vanillic acid), chalcones (cardamonin), coumarins (herniarin, coumarin), organic acids (succinic acid, malic acid), fatty acids (palmitic acid, stearic acid, dodecanoic acid), sterols, carotenoids, resins, polysaccharides, tannins and lignans [156, 157].

Traditionally, *A. absinthium* has been used to treat digestive disorders, helminthiasis, anemia, anorexia, sleeplessness, bladder illnesses, microbiological disease, hepatic diseases, ulcers, and fever. Today anticarcinogenic, hepatoprotective, anti-inflammatory, antioxidant, immunomodulatory, cytotoxic, analgesic, neuroprotective, and anti-depressant effects of this plant have been identified [158]. Rezaie and colleagues evaluated the sedative, pre-anesthetic, and anti-anxiety effects of methanol and chloroform extracts of Artemisia. When compared to diazepam, artemisia extract (100, 200, and 400 mg/kg B.W) significantly reduced anxiety, induction time, and increased sleeping time in rats [159]. Rakhshandeh *et al.* studied the hypnotic effect of *A. absinthium* and its fractions in rats under pentobarbital sedation. The duration of sleep was lengthened by *A. absinthium* extract (100, 200 mg/kg), aqueous, ethyl acetate, and n-butanol fractions (200 mg/kg) (Table **1**). Also, *A. absinthium* extract, aqueous, and ethyl acetate fractions reduced sleep latency, most likely by modulating the GABAergic system (Table **1**) [160]. One of the biological functions of Artemisia alkaloids is to induce sleep. Artemisia species produce tryptophan as a secondary metabolite, which works as a natural sedative drug and is responsible for manufacturing numerous tryptophan-derived metabolites. In addition, it is a precursor in plants' production of indole alkaloids, melatonin, and serotonin (a neurotransmitter that regulates sleep, mood, and appetite) [161].

### 3.21 *Lagenaria vulgaris* and *Cucurbita pepo*

*Lagenaria vulgaris*, also known as *Lagenaria siceraria* (Molina) Standley, calabash, and bottle gourd, is a member of the Cucurbitaceae family that grows on almost every continent [162]. *Cucurbita pepo* (Field pumpkin), another member of the Cucurbitaceae family, is a Persian plant that has been prescribed for the treatment of insomnia [13]. The Cucurbitaceae family's plants are rich in phytochemicals such as terpenoids, glycosides, alkaloids, saponins, tannins, steroids, carotenoids, and resins, among many others, which are found in the leaves, stems, flowers, fruits, seeds, and roots of plants. These herbs' constituents exhibit pharmacological properties such as hypolipidemic, antihyperglycemic, anticancer, antimicrobial, analgesic, anti-inflammatory, anti-stress, immunomodulatory, and sedation [163-165]. According to studies, the fruit of *L. siceraria* (Molina) Standley has antioxidant and radical scavenging action that can help in the treatment of the mental condition [166, 167]. Rahimi *et al.* demonstrated that macerated and soxhlet extract fruit of *Cucurbita pepo* (200 mg/kg) enhanced pentobarbital-induced sleep duration, and fruit (200 mg/kg), seed (50 and 100 mg/kg), and fractions of *Lagenaria vulgaris* (water, ethyl acetate, and n-butanol) increased sleeping time and sleep length in mice, as did diazepam (Table **1**) [168]. The hypnotic effects of *L. vulgaris* and *C. pepo* are exerted probably through GABA receptors. It has been shown that flavone glycosides isolated from *L. vulgaris,* such as vitexin, isovitexin, isoorientin, lutonarin, and saponarin interact with GABA_A_ receptors and present a hypnotic effect (Table **1**) [162, 169].

### 3.22 *Capparis spinose*

*Capparis spinose* (Capparaceae), generally known as Caper, is a Mediterranean shrub found from the Atlantic coast to the Black Sea, Crimea, and Armenia, as well as the east side of the Caspian Sea and Iran [170]. Polyphenols, flavonoids, alkaloids, and tannic acid are abundant in several sections of *C. spinosa*, including fruits and roots, which have been used as a traditional herbal in the treatment of liver and kidney ailments, paralysis, diabetes, splenomegaly, hemorrhoids, ulcers, rheumatoid arthritis, and mental problems [171, 172]. *C. spinosa* hydro-ethanolic extract (100 and 300 mg/kg) reduced neuroinflammation in the LPS-induced inflammation in the microglia *of rats and has a neuroprotective impact [**173**].* Rakhshandeh and colleagues demonstrated that a hydro-alcoholic extract of *C. spinose* (30, 60, and 120 mg/kg) and its fractions (n-hexane, water, and ethyl acetate fractions) substantially enhanced sleeping duration in pentobarbital-induced rats compared to diazepam (Table **1**) [174]. In another study, aqueous extract (100 and 200 mg/kg), methanolic extract and fraction (100, 200, and 400 mg/kg), and dichloromethane (25, 50, and 100 mg/kg) fraction of *C. spinosa* reduced the total distance movement and increased the sleeping time in pentobarbital induced sleep model (Table **1**). In this study, dichloromethane had the highest sedative effects, which seems non-polar agents involving opioid receptors are responsible for the hypnotic effects [175].

### 3.23 *Brassica oleracea*

Red cabbage (*Brassica oleracea* L; *Brassicaceae*) is a popular food in Asia and Europe due to its low calorie-high fiber content [176]. It is also a rich source of anthocyanins, vitamin C, tocopherol, glucosinolates, alkaloids, saponins, tannins, phlobatannins, terpenoids, flavonoids, glycosides, and steroids [177, 178]. *Brassica* consumption has been linked to a lower risk of common malignancies. Recently, the preventative impact of these herbs on cardiovascular disease, hypercholesterolemia, oxidative stress and longevity, neurological disorders, and diabetic nephropathy has been shown [179-185]. Hosseini *et al.* demonstrated the influence of a red cabbage hydro-alcoholic extract on mouse sleeping behavior. They discovered that the extract and its fractions (ethyl acetate, n-butanol, and aqueous fractions) at 50-200 mg/kg enhanced sleep duration at levels equivalent to diazepam. The extract and solely the ethyl acetate fraction were shown to reduce sleep latency. In this study, the ethyl acetate fraction exhibited a more significant hypnotic effect than the other two fractions, indicating that intermediate polarity elements such as flavonoids are responsible for red cabbage's sleep-prolonging consequences [186]. The potential of flavonoids like quercetin, kaempferol, caffeic acid, and ferulic acid in the brain has been demonstrated *via* two primary mechanisms: inhibition of oxidative stress and neuroinflammation. Flavonoids can reduce reactive oxygen species (ROS) formation and lipid peroxidation in rats' brains. They can also inhibit inflammatory and pro-inflammatory cytokines in the brain [187].

### 3.24 *Portulaca oleracea*

*Portulaca oleracea* (Purslane) is a worldwide herbaceous annual succulent plant of the *Portulacaceae* family that grows in the warm regions of the United States, Europe, the Mediterranean, and Asia [188-196]. It contains higher omega-3 fatty acids, making it appropriate for improving brain and cardiovascular system performance [189-197]. Moneim's study underlined purslane's anti-apoptotic activity in the midbrain and striatum and its potential for preventing brain damage and neurodegenerative disorders caused by oxidative stress [198]. In a study, the three doses of 25, 50, and 75 mg/kg of Purslane decoction extract increased the sleeping time of mice (Table **1**) [199]. Purslane hydro-alcoholic extract (25, 50, 75, and 100 mg/kg) and its fractions (25 mg/kg) prolonged the duration of pentobarbital-induced sleep in rats compared to diazepam. The hydro-alcoholic extract and n-butanol fraction reduced sleep latency, indicating that low polar agents such as alkanes, sterols, and terpenoids possibly manifested hypnosis in research by Hamedi *et al.* [200]. Isoquinoline alkaloids from *Portulaca*, such as catechol isoquinolines, have been shown in studies to possess α2-adrenergic receptor agonist action, which can be utilized to treat sleep problems [201].

### 3.25 *Cuscuta epithymum*

*Cuscuta epithymum* is an annual, occasionally perennial parasitic genus of the Convolvulaceae family that dies if the seedling does not identify a host once the seedling's nutrition reserve is depleted [202]. Some of the chemical constituents of *C. epithymum* are chlorogenic acid, hyperoside, astragalin, kaempferol, quercetin, obtucifoliol, cycloartanol, cycloeucalenol, and sterols [203]. *C. epithymum* has long been used locally and traditionally in various regions. It was mentioned in several Persian medicine references and in India for treating disorders such as kidney and liver, joint, urinary tract, immune system, gastrointestinal tract, and nervous system [203]. Several studies have examined its sedative and hypnotic properties. Forouzanfar *et al.* revealed that the hydroalcoholic extract of *C. epithymum* and its fractions (water, ethyl acetate, and n-butanol fraction) could have sedative-hypnotic effects in mice probably through GABAergic System (Table **1**) [204]. Also, Taleghani *et al.* revealed that μ-opioid and GABA_A_ receptor antagonists could reduce the anti-nociceptive activity of *C. epithymum* Murr. extract in male mice [205], suggesting this plant may exert its effects on the nervous system through the opioidergic and GABAergic systems.

### 3.26 *Perovskia abrotanoides* Karel

*Perovskia abrotanoides* Karel, a member of the Lamiaceae family, is mainly grown along the edges of mountainous in the dry and cold climates of Iran, Northern Pakistan, and Northwestern India [206, 207]. Most components in the plant are terpinolene, ursolic acid, stigmasterols, betulinic acid verbenone, cirsimaritin, sabinene, terpinen and terpinen-4-ol [208, 209]. This plant is used chiefly as a fortifier, rheumatic pains, anti-inflammatory, antiinfective, and sedative [208]. Forouzanfar *et al.* discovered that treated mice with hydro-alcoholic extract of *Perovskia abrotanoides* Karel. at doses ranging from 25-200 mg/kg and n-butanol fraction (25 and 50 mg/kg) increased sleep duration and decreased sleep latency in a manner similar to diazepam, most likely *via* the GABAergic system (Table **1**) [210]. Each component has antagonistic binding potential to GABA_A_ receptor sites, resulting in enhanced efficiency of the leading GABA site and, as a result, an increase in chloride channels and membrane hyperpolarisation. Binding interactions and receptor activation often induce anxiolytic, sedative, and hypnotic activities [211].

### 3.27 *Tanacetum parthenium*

*Tanacetum parthenium* (feverfew) is a perennial herbaceous plant of the Asteraceae family with a wide range of existence in Asia, Europe, and America. It is spread in Iran's northern, western, eastern, and central areas [212]. Essential oil of feverfew contains compounds such as tanetin, santin, camphor, ρ-cymene, chrysanthenyl acetate, farnesol, palmitic acid, myristic acid, cinnamic acid, sesquiterpene lactones which contribute to the anti-inflammatory, antioxidant, antimicrobial, cytotoxicity properties of feverfew [213-215]. In addition, the α-pinene compounds may have sedative and anxiety-relieving properties [216]. The neuroprotective and sedation effects have been reported in some studies. Moscano *et al.* found that *T. parthenium,* in combination with magnesium, riboflavin, and CoQ10 decreased headache occurrence and pain intensity in children and adolescents suffering from tension-type headaches and migraine, both of which are chronic neurological illnesses [217]. Also, Forouzanfar *et al.* showed that hydro-alcoholic extract of Feverfew (50-200 mg/kg) and ethyl acetate fraction (50 mg/kg) improved insomnia in mice-induced sleeping behaviors by pentobarbital (Table **1**) [218]. The results of studies showed that *T. parthenium,* probably by acting on the GABAergic system, exerted hypnotic, anxiolytic, and antidepressant-like effects because the antagonist of receptor GABA reversed these effects (Table **1**) [218, 219].

### 3.28 *Solanum lycopersicum*

*Solanum lycopersicum* L. (Cultivated tomato-Solanaceae family), as a nutritional supplement cultivation plant in human nutrition, contains a wide range of health-promoting bioactive compounds, including carotenoids (lycopene, β-carotene), vitamins C and E, anthocyanins (petunidin and malvidin), and polyphenols compounds [220]. It has excellent nutritional value and antioxidant activity, which adds to the fruits' pharmacological features, such as reducing the formation of reactive species, cardiovascular disease, neurological illnesses, and some kinds of cancer [221]. Plants of the Solanaceae family, sometimes known as nightshades, are exceptionally high in alkaloids. Alkaloids exert numerous neuroprotective and stimulating effects on the CNS in various conditions, such as epilepsy, psychiatric disorders, Alzheimer's disease, Huntington's disease, schizophrenia, cerebral ischemia, depression, anxiety, and others [78, 222]. In this way, Molkara *et al.* indicated that hydro-alcoholic extract of *S. lycopersicum* and *S. nigrum,* by increasing the sleep duration and decreasing sleep latency, exerts a hypnotic effect in sleep-induced mice probably through positive allosteric regulation of the GABA_A_ receptor complex [223]. Furthermore, *S. lycopersicum* L. has more GABA- a non-proteinogenic amino acid with hypotensive effects- than other crops. Glutamate decarboxylase (GAD) is a crucial enzyme in the production of GABA found in the tomato genome (Figs. **1** and **2**) [224].

### 3.29 *Rosa damascena*

*Rosa damascena* (Rose or Gul-e-Surkh) belongs to the Rosaceae family plant that originated in the Middle East but is now grown all over the world and used for fragrance, medicine, and the food industries [225]. Rose extracts have compelling free radical scavenging activities (when compared to other plants), which are connected with the level of phenolic compounds (Fig. **2**) [226]. *Rosa damascena* has been found to act on the central nervous system. Studies revealed *Rosa damascena* flowers have antimigraine and antiepileptic effects [227, 228]. Keyhanmehr *et al.* discovered that breathing *Rosa damascena* essential oil for two weeks reduced sleep resistance, difficulties getting up in the morning, nightmares, and waking up throughout the night in children with sleep disorders [229]. According to Sanatkaran *et al.* there is no significant effect of aromatherapy with red rose essential oil and lavender on the sleep quality of mentally and physically healthy female students for seven nights, which is likely owing to the short treatment period [230]. In addition, in a meta-analysis of randomized controlled studies, the administration of *Rosa damascena* was identified as a viable complementary and alternative medicine strategy for improving adults' sleep quality [231]. The ethanolic and aqueous extracts of *R. damascena* in dosages of 500 and 1000 mg/kg considerably enhanced the pentobarbital-induced sleeping period in comparison to diazepam and chloroform extract and had no hypnotic effect. [232]. Rakhshandah *et al.* showed the hypnotic effect of ethanol extract and its aqueous, ethyl acetate, and n-butanol fractions (250 and 500 mg/kg) of *R. damascena* through prolonged sleeping time that was more prominent in ethyl acetate fraction [233]. Some components of *R. damascena*, such as flavonoids, geraniol, saponin, and eugenol, have been demonstrated to exhibit anxiolytic action and contribute to the hypnotic effect of this plant *via* benzodiazepine receptors (Table **1**, Fig. **1**) [234].

### 3.30 *Crocus sativus*

*Crocus sativus* L. (Iridaceae) is widely farmed in Iran and other countries such as India and Greece [235-237]. *C. sativus* chemical constituents include carbohydrates, proteins, amino acids, minerals, mucilage, vitamins (especially riboflavin and thiamine), anthocyanin, lycopene, zeaxanthin, flavonoid, starch, gums, and other chemicals. Crocin, crocetin, and the monoterpene aldehydes, including picrocrocin and safranal, are saffron's principal bioactive chemicals [238, 239]. Based on animal and *in vitro* research, modern medicine has shown that saffron possesses chemotherapy-protecting, anti-inflammatory, antioxidant, and anti-toxicant properties. Saffron's neuroprotective benefits have been studied for their ability to reduce symptoms of neuropsychiatric and neurodegenerative conditions [240, 241]. Saffron ethanolic extract (5 and 10 µg/rat) improves learning and memory deficits and restores oxidative stress indicators in the hippocampus of multiple sclerosis experimental animals [242]. Furthermore, ethanolic and aqueous extracts (50,100, and 200 mg/kg, i.p.) and safranal (0.025, 0.05, and 0.1 mg/kg, i.p.) decreased neuropathic pain in rats in a dose-dependent manner (Table **1**) [243]. It has been suggested that the major active components of saffron, safranal, and crocin are responsible for its depressive and anxiolytic properties, which may be mediated through the GABA(A)-benzodiazepine receptor complex (Table **1**) [244, 245]. The administration of the aqueous saffron extract (0.56 g/kg) and safranal (0.15 and 0.35 mL/kg) show anxiolytic and hypnotic effects in mice [246]. In a trial on diabetic patients, Shahdadi *et al.* discovered that taking a saffron capsule (300 mg daily) for a week reduced anxiety and improved sleep quality (Table **2**) [247]. Lopresti *et al.* demonstrated that saffron extract (14 mg twice daily) enhanced sleep quality during one month in healthy people with self-reported sleep issues (Table **2**) [248]. Pachikian *et al.* also discovered that saffron extract (15.5 mg daily for six weeks) increased the ability to fall asleep, sleep quality, sleep latency, sleep length, body pain, physical, and emotional limitation scores in subjects with mild to severe sleep problems linked with anxiety [249].

### 3.31 *Lawsonia inermis*

*Lawsonia inermis* Linn from the Lythraceae family, sometimes known as henna, is utilized throughout the world. Many alkaloids, phenolics, flavonoids, tannins, saponins, carbohydrates, proteins, fat, ash, crude fiber, terpenoids, quinones, coumarins, and resins have been identified from *L. inermis* [250, 251].

The central nervous system features of *L. inermis* have been studied. Its extract has been investigated for its sedative and neuropathic pain-relieving properties [251, 252]. The crude ethanolic extract of *L. inermis* at doses of 0.25-2.0 g/kg and 2-hydroxy-1,4-naphthoquinones (lawsone) obtained from the chloroform extract substantially extended pentobarbitone-induced sleeping time in rats [253]. The ethanolic extract of *L. inermis* flowers (500 mg/kg) demonstrated considerable muscular relaxation and reduction in the start and prolonging of sleep duration produced by pentobarbitone (Table **1**) [254]. It is conceivable that *L. inermis* extracts work by potentiating GABAergic inhibition in the CNS *via* membrane hyperpolarization, resulting in a reduction in the cortical neurons firing rate in the brain or by directly activating GABA receptors [255].

### 3.32 *Ziziphus jujube*

*Ziziphus jujuba* Mill, often known as jujube, is a tiny, edible, date-like fruit plant from the Rhamnaceae family that is native to Asia (China, India, Iran), Southern Europe, North Africa, and Middle Eastern nations [256]. One of the primary functions of jujube was thought to be neuroprotection by inducing neurons outgrowth and neurotrophic factors expression *via* cAMP-dependent PKA signaling, anti-oxidation activity *via* enhancing cellular Nrf2-dependent ARE-driven gene expressions, improving choline acetyltransferase (ChAT) activity, increasing the level of acetylcholine (ACh) *via* inhibition of acetylcholinesterase, and stimulates the expression of GABA receptor subunits [257]. It benefits our brain by relaxing the mind and boosting sleep quality. In a placebo-controlled trial, total sleep duration, sleep quality, and sleep latency of chronic insomnia individuals were improved following treatment with a capsule of *Ziziphus spinosa* (2 g/day) (Table **2**) [258]. Mahmoudi *et al.* showed that treatment of postmenopausal women with a 250 mg oral jujube seed capsule improved their sleep quality in 21 days (Table **2**) [259]. Flavonoids, saponin, phenolics, cyclopeptide alkaloids, and jujuboside A and B might be the bioactive substances responsible for these biological effects [260].

### 3.33 *Passiflora incarnata*

The genus *Passiflora* has 500 species that are typically found in warm and tropical climates. *Passiflora incarnata* (Passifloraceae) is the most well-known species in this genus [261]. The flowers, leaves, and seeds of *P. incarnata* contain a variety of bioactive components such as alkaloids, indole alkaloids having the β-carboline ring (harman, harmine, harmalol, and harmaline), steroid, β-sitosterol, phenols, glycosyl flavonoids (vitexin, isovitexin, orientin, and chrysin) and cyanogenic substances [262]. Previous research indicates that *P. incarnata* has been widely used to treat sedatives, anxiety, and sleep. The leaves and flowers of *P. incarnata* have been shown to have CNS-depressant and sleep-inducing properties [262]. Kim *et al.* discovered that after a single (500 mg/kg) or repeated (250 mg/kg) oral administration of *P. incarnata* L. in mice, immobility time, palpebral closing time, and blood melatonin levels were significantly increased (Table **1**), and mRNA expression levels of GABA receptors were decreased considerably in C6 rat glioma cells treated with *P. incarnata* L. [263]. In addition, calretinin (calcium-binding protein) released by GABAergic neurons in the hippocampus and hypothalamus, as well as serum melatonin and serotonin, were found to be increased in mice treated with ethanol extracts of *P. incarnata* (500 mg/kg) (Table **1**) [264]. In placebo-controlled research, drinking a cup of *P. incarnate* herbal tea improved sleep for healthy persons with modest changes in sleep quality (Table **2**) [265]. Furthermore, in another study, *an extract of P. incarnata (500 mg/kg) enhanced sleep duration and slow-wave sleep (SWS) while reducing sleep latency by preventing rapid eye movement (REM) in rats* (Table **1**) [266].

## CONCLUSION

Insomnia is one of the most common sleep disorders worldwide, and in Iran, it is defined by sleep problems that affect the routine activities and decrease life quality. To prevent the adverse effects of synthetic medicines used to treat insomnia, particular attention has lately been paid in Iran to herbal therapies as alternatives to synthetic medicines. People utilize herbal remedies more for mild/moderate disorders, beginning therapy before using conventional medicine, and less for preventing illnesses, boosting health, and treating severe illnesses. Dissatisfaction with conventional therapy, previous positive experiences, and family traditions are other reasons why herbal medicine is favored as treatment [267].

Ample research has justified the acceptable reason and relevance of the use of these herbs in the treatment of insomnia. It is worth noting that in this study, we looked into various Persian herbs in a clinical trial and *in vivo* to treat insomnia, such as *Artemisia annua*, *Salvia reuterana*, *Viola tricolor*, *Passiflora incarnata*, lettuce, and *Capparis spinose*, to mention a few (Fig. **1**). According to research, herb extracts and fractions, particularly n-butanol fractions with non-polar agents, impact the benzodiazepine receptors and have hypnotic properties. Also, alkaloids, glycosides, flavonoids, saponins, and tannins in practically every plant are mentioned making them the popular natural compounds to help with sleep disorders and promote calmness.

One of the primary functions of jujube was thought to be neuroprotection by inducing neurons outgrowth and neurotrophic factors expression *via* cAMP-dependent PKA signaling, anti-oxidation activity *via* enhancing cellular Nrf2-dependent ARE-driven gene expressions, improving ChAT activity, increasing the level of ACh *via* inhibition of acetylcholinesterase, and stimulates the expression of GABA receptor subunits [257]. The presence of compounds with anti-acetylcholinesterase activity in *A. vera* can partly explain the observed changes in sleep impairment. *S. reuterana* components likely induced sedation by inhibiting acetylcholinesterase or contacting GABA receptors [111].

GABAA receptors are key inhibitory neurotransmitter receptors in a variety of neuropsychiatric disorders. GABAA receptors can be activated and regulated by a variety of medicines. Diazepam and other benzodiazepines are well-known medications that operate as positive allosteric modulators of a subset of these receptors. According to Sakurai *et al.*, the sedative action of coriander leaf extract is attributable to the hyperactivity of inhibitory neurons in the brain because the leaf extract (600 mg/kg) raised the gene expression of the GABA-A receptor-1 subunit and decreased the gene expression of GABA transporter [59]. Linalool, the primary coriander component, has various neuropharmacological effects such as anti-anxiety, sedative, and anticonvulsant [54]. Other monoterpenoids such as limonene, myrcene, γ-terpinene, and α-pinene in *C. sativum* are shown to possess sedative and anxiolytic effects due to the interaction of monoterpenes with GABAA receptors [55, 56]. Furthermore, the *C. aurantium* L. mechanism on the CNS can be related to flavonoid glycosides (naringin, hesperidin, and neohesperidin, flavones, flavonones, polimethoxylates, glycan peptides) existing in it. In addition, Fernandez *et al.* detected the anxiolytic and sedative effects of a range of flavonoid glycosides (myrcitrin, naringin, and gossypin) in mice [44].

*Lactuca sativa* seed extract contains caftaric acid, chlorogenic acid, and chicoric acid, significant antioxidant phenolics that protect against oxidative stress produced by sleep disruption [65]. These findings indicate that lactucin and lactucopicrin, which are found in green romaine leaf extract, bind efficiently to GABAA receptors and serve as the active chemical that induces sleep [66]. Also, the n-butanol fraction (400 mg/kg) of *Lactuca serriola* aerial parts caused a sedative effect among the other fractions but not more than the hydro-alcoholic extract, suggesting that the active molecules responsible for the impact of lettuce are non-polar agents such as sterols, alkanes, and specific terpenoids [74]. According to studies, terpenoids with diverse chemical structures exhibit varying affinities for the GABA receptor and modify GABA receptors either by contact with a common BZD site on the receptor or independently of BZD sites [75, 76].

Alnamer *et al.* demonstrated that methanolic (200, 400, and 600 mg/kg) and aqueous (200 and 400 mg/kg) extracts of *Lavandula officinalis* L. had sedative and hypnotic effects in mice when compared to diazepam, which was mediated through the GABAergic system [82]. Furthermore, for four weeks, lavender aromatherapy improved sleep quality, quantity, mood, and life quality in diabetic patients with sleeplessness [83]. Orange peel essential oil has active ingredients such as linalyl acetate and linalool that have been reported to have narcotic effects through interaction with the GABA receptor [100]. Also, hesperidin, the main flavonoid in *C. sinensis*, was identified as the active principle in this plant responsible for sedation [101].

As a result, it can be inferred that the active ingredients of *P. eldarica* responsible for sleep prolongation include low polar agents in an n-butanol fraction, such as alkanes, sterols, and terpenoids [105]. Also, studies showed that α-pinene, and 3-carene, monoterpene of the *Pinaceae, improved sleep quality through* binding to the BZD site of α_1_ and γ_2_ subunits of GABA_A_-BZD receptor [106, 107]. Similarly, pretreatment of animals with compounds from ethanol extract of the *S. leriifolia* (10, 15, and 20 mg/kg) caused a significant seductive and muscle relaxant-like effect through interaction with GABAA receptor similar to that of BDZ [116]. Furthermore, valerian is known to stimulate the release of neurotransmitters such as GABA and inhibit the enzyme-induced breakdown of GABA in the brain, perhaps acting as a precursor for GABA synthesis. However, it has been discovered that valerian lignan hydroxy pinoresinol binds to benzodiazepine receptors [123, 128]. Moreover, some of the chemicals in *V. tricolor*, such as rutin, have been shown in studies to alleviate anxiety by activating the GABAergic system in the basolateral amygdala and may also enhance the GABAergic systems [138].

Rakhshandeh *et al.* studied the hypnotic effect of *A. absinthium* and its fractions in rats under pentobarbital sedation. The sleep duration was lengthened by *A. absinthium* extract (100, 200 mg/kg), aqueous, ethyl acetate, and n-butanol fractions (200 mg/kg). Also, *A. absinthium* extract, aqueous, and ethyl acetate fractions reduced sleep latency, most likely by modulating the GABAergic system [160]. Additionally, *Artemisia* species produce tryptophan as a secondary metabolite, which works as a natural sedative drug and is responsible for manufacturing numerous tryptophan-derived metabolites. In addition, it is a precursor in plants' production of indole alkaloids, melatonin, and serotonin [161].

Rahimi *et al.* demonstrated that macerated and soxhlet extract fruit of *Cucurbita pepo* (200 mg/kg) enhanced pentobarbital-induced sleep duration, and fruit (200 mg/kg), seed (50 and 100 mg/kg), and fractions of *Lagenaria vulgaris* (water, ethyl acetate, and n-butanol) increased sleeping time and sleep length in mice, as did diazepam [168]. The hypnotic effects of *L. vulgaris* and *C. pepo* are exerted probably through GABA receptors. It has been shown that flavone glycosides isolated from *L. vulgaris,* such as vitexin, isovitexin, isoorientin, lutonarin, and saponarin interact with GABAA receptors and present a hypnotic effect [162, 169]. Also, it affects the CNS and treats several neurological conditions. Several experiments were conducted to examine the antidepressant efficacy of *O. basilicum* L. extract. Researchers discovered that the methanolic extract of *O. basilicum*, due to its antioxidative potency and free radical scavenging activity, attenuates the depressant-like actions against oxidative damage in rats [153]. Askari *et al.* discovered that hydro-alcoholic extract of *O. basilicum* (25, 50, or 100 mg/kg), ethyl acetate (50 mg/kg), n-butanol (50 mg/kg), and aqueous fractions (50 mg/kg) increased sleep duration and, while sleep latency was significant in hydro-alcoholic and n-butanol fractions [154]. The possible mechanism for sedative-hypnotic effects of *O. basilicum* could be associated ‎with‎ the‎ presence ‎of‎ non-polar agents such as linalool, eugenol, bergamotene, germacrene D, cadinene, cadinene, selinene, and spathulenol identified in the extracts of *O. basilicum* [155].

Hydroalcoholic extract of *C. epithymum* and its fractions (water, ethyl acetate, and n-butanol fraction) could probably have sedative-hypnotic effects in mice through GABAergic System [204]. Also, Taleghani *et al.* revealed that μ-opioid and GABA_A_ receptor antagonists could reduce the anti-nociceptive activity of *C. epithymum* Murr. extract in male mice [205], suggesting this plant may exert its effects on the nervous system through the opioidergic and GABAergic systems. Aqueous extract (100 and 200 mg/kg), methanolic extract and fraction (100, 200, and 400 mg/kg), and dichloromethane (25, 50, and 100 mg/kg) fraction of *C. spinosa* reduced the total distance movement and increased the sleeping time in pentobarbital induced sleep model. In this study, dichloromethane had the highest sedative effects, which seems non-polar agents involving opioid receptors are responsible for the hypnotic effects [175]. The hydro-alcoholic extract and n-butanol fraction of purslane reduced sleep latency, indicating that low polar agents such as alkanes, sterols, and terpenoids possibly manifested hypnosis in research by Hamedi *et al.* [200]. Isoquinoline alkaloids from purslane, such as catechol isoquinolines, have been shown in studies to possess α_2_-adrenergic receptor agonist action, which can be utilized to treat sleep problems [201].

Forouzanfar *et al.* discovered that treated mice with hydro-alcoholic extract of *Perovskia abrotanoides* Karel. at doses ranging from 25-200 mg/kg and n-butanol fraction (25 and 50 mg/kg) increased sleep duration and decreased sleep latency like diazepam, most likely *via* the GABAergic system [210]. Each component has antagonistic binding potential to GABA_A_ receptor sites, resulting in enhanced efficiency of the leading GABA site and, as a result, an increase in chloride channels and membrane hyperpolarisation. Binding interactions and receptor activation often result in anxiolytic, sedative, and hypnotic activities [211]. Hydro-alcoholic extract of *S. lycopersicum* and *S. nigrum,* by increasing the sleep duration and decreasing sleep latency, exerts a hypnotic effect in sleep-induced mice, probably through positive allosteric regulation of the GABA_A_ receptor complex [223]. Glutamate decarboxylase is a crucial enzyme in producing GABA found in the tomato genome [224].

Some components of *R. damascena*, such as flavonoids, geraniol, saponin, and eugenol, have been demonstrated to exhibit anxiolytic action and contribute to the hypnotic effect of this plant *via* benzodiazepine receptors [234]. In addition, it has been suggested that the primary active components of saffron, safranal, and crocin are responsible for their depressive and anxiolytic properties, which may be mediated through the GABA(A)-benzodiazepine receptor complex [244, 245]. Similarly, it is conceivable that *L. inermis* extracts work by potentiating GABAergic inhibition in the CNS *via* membrane hyperpolarization, resulting in a reduction in the cortical neurons firing rate in the brain or by directly activating GABA receptors [255].

Amino acids in protein-rich almonds are essential in their sedative/hypnotic properties. As an endogenous neurotransmitter, glycine affects the CNS [28]. Serine and glycine have been associated with hypnotic effects. Also, glycine improves sleep quality in humans who complain of sleep disorders. Phenylalanine and tyrosine have shown positive effects on sleep patterns. Furthermore, it has been demonstrated that the motherwort extracts with glycine, valine, and arginine reduced anxiety in animals [89]. According to research, the neurological mechanism of action of *Leonurus cardiaca* primarily depends on its interaction with the GABA site of the GABA type A receptor [90].

## Figures and Tables

**Table 1:** Experimental evidence of the effects of Persian herbal treatments on insomnia.

Plant	Dose/Route	Study Design	Mechanism	Outcomes	References
*A. vera*/aqueous extract of leaves	50, 100 and 200 (mg/kg), i.p.	In rats, on pentobarbital-induced sleeping	↑ Acetylcholine ↓ Choline-esterase	↑ NREM ↓ REM	[268]
*A. vera*/aqueous extract of leaves	200 (mg/kg), i.p.	In rats, on pentobarbital-induced sleeping	↑ Acetylcholine ↓ Choline-esterase	Sedative-hypnotic effects prolonged loss of righting reflex	[268]
*A. vera*/aqueous extract of leaves	100 and 200 (mg/kg), i.p.	In rats, on pentobarbital-induced sleeping	↑ Acetylcholine ↓ Choline-esterase	↓ Locomotion activity	[268]
*Amygdalus communis*/ extract	100, 200, 400 (mg/kg), i.p.	In rats, pentobarbital-induced sleeping	An endogenous neurotransmitter, glycine, affects the CNS	Sedative-hypnotic effects ↑ NREM ↓ REM	[27]
*Amygdalus communis*/ extract	400 (mg/kg), i.p.	In rats, the extract was injected 30 min before pentobarbital (40 mg/kg)	An endogenous neurotransmitter, glycine, affects the CNS	↑ Sleep duration ↓ Locomotion activity ↑ NREM ↓ REM	[27]
*Artemisia annua*/ essential oil	470 (mg/kg), i.p.	In rats, the injection was done 30 min before sodium pentobarbital (40 mg/kg)	BZD receptors	↑ Immobility time ↓ Activities ↑ Sleep duration ↓ Sleep latency	[34]
*Artemisia annua*/crude ethanolic extract	450 (mg/kg), i.p.	In rats, the injection was done 30 min before sodium pentobarbital (40 mg/kg)	BZD receptors	↑ Immobility time ↓ Activities prolonged the sleeping time and lost latency	[34]
*Artemisia annua*/Methanol extract was partitioned into chloroform, petroleum ether, and ethyl acetate	50, 100, and 200 (mg/kg), i.p.	In mice, flumazenil (3 mg/kg, i.p.) injected 15 min before chloroform fraction (200 mg/kg).	*via* BZD receptors pathways	↑ Immobility time sedative effects	[37]
*Citrus aurantium*/ flowers aqueous extract	62.5 and 125 (mg/kg), i.p.	In rats, 30 min after extract injection, animals received sodium pentobarbital (20 mg/kg, i.p)	Through binding of flavonoids to the GABA-A receptors	↑ Percentage of time spent in the open arms ↓ In closed arms ↓ Locomotor activity Sedative effects	[41]
*Citrus aurantium/* flowers aqueous extract	125 and 250 (mg/kg), i.p.	In rats, 30 min after extract injection, animals received sodium pentobarbital (20 mg/kg, i.p)	Through binding of flavonoids to the GABA-A receptors	↑ Sleeping time ↓ Onset of sleep sedative effects	[41]
*Citrus aurantium*/ essential oil	1.0 (g/kg), p.o.	In mice, sodium pentobarbital-induced sleep (40 mg/kg, i.p.)	Through binding of flavonoids to the GABA-A receptors	↑ Sleeping time	[40]
*Coriandrum sativum/* aqueous extract	200, 400 and 600 (mg/kg), i.p.	In mice, extracts were injected 30 minutes before pentobarbital injection (40 mg/kg).	Interaction of monoterpenes with GABA_A_ receptors	↑ Sleeping time sedative-hypnotic effects	[51]
*Coriandrum sativum/* hydro-alcoholic extract	400 and 600 (mg/kg), i.p.	In mice, extracts were injected 30 min before pentobarbital injection (40 mg/kg)	Interaction of monoterpenes with GABA_A_ receptors	↑ Sleeping time sedative-hypnotic effects	[51]
*Coriandrum sativum* L. seeds/ essential oil	600 (mg/kg), i.p.	In mice, extracts were injected 30 minutes before pentobarbital injection (40 mg/kg).	Interaction of monoterpenes with GABA_A_ receptors	↑ Sleeping time sedative-hypnotic effects	[51]
*Coriandrum. Sativum*/ seed extract	250, 500 and 1000 (mg/kg), p.o.	In mice, pentobarbital-induced sleep (50 mg/kg, i.p.)	Interaction of monoterpenes with GABA_A_ receptors	Failed to exert effects on sleep onset and duration	[52]
*Coriandrum sativum* seeds/essential oil	8.6 and 86 (μg), intracerebroventricular injection	In neonatal chicks	Interaction of monoterpenes with GABA_A_ receptors	Sedative effect	[53]
*Coriandrum sativum/* leaf extract	600 (mg/kg), p.o.	In mice, Administration of pentobarbital (21.6 mg/kg. i.p.) 2 h after receiving extracts	Interaction of monoterpenes with GABA_A_ receptors	Sedative action	[59]
*Lactuca sativa/* hydroalcoholic extract	400 (mg/Kg), i.p.	In mice, extracts were administered 30 minutes before pentobarbital injection (i.p.)	Bind efficiently to GABAA receptors	↑ Sleep duration ↓ Sleep latency	[64]
*Lactuca sativa/* n-butanolic extract	200 (mg/Kg), i.p.	In mice, extracts were administered 30 minutes before pentobarbital injection (i.p.)	Bind efficiently to GABAA receptors	↑ Sleep duration ↓ Sleep latency	[64]
*Lactuca serriola* aerial parts*/*hydro-alcoholic extract	50-400 (mg/kg), i.p.	In mice, extract, and its fractions were injected 30 min before administration of pentobarbital	Modify GABA receptors	↑ Sleeping time ↓ Sleep latency	[269]
*Lactuca serriola* aerial parts/ n-butanolic extract	400 (mg/kg), i.p.	In mice, extract, and its fractions were injected 30 min before administration of pentobarbital	Modify GABA receptors	Sedative effect	[269]
*Lavandula officinalis*/ Methanolic extract	200, 400, and 600 (mg/kg), p.o.	In mice, the activity of extract on the CNS was then studied using a battery of behavioral tests	Through the GABAergic system	Sedative-hypnotic effects	[82]
*Lavandula officinalis*/ aqueous extract	200 and 400 (mg/kg), p.o.	In mice, the activity of extract on the CNS was then studied using a battery of behavioral tests	Through the GABAergic system	Sedative-hypnotic effects	[82]
*Nepeta glomerulosa/* hydro-alcoholic extract	50-200 (mg/Kg), i.p.	In mice, the extract was injected 30 min before administration of pentobarbital (30 mg/kg, i.p.)	*Via* the GABAergic system	↑ Sleep duration ↓ Sleep latency	[95]
*Nepeta glomerulosa/* n-butanol extract	50 and 100 (mg/Kg), i.p.	In mice, the extract was injected 30 min before administration of pentobarbital (30 mg/kg, i.p.)	*Via* GABAergic system	↑ Sleep duration ↓ Sleep latency	[95]
*Pinus eldarica* hydroalcoholic/extract	25-200 (mg/kg), i.p.	In mice, extracts were injected 30 min before administration of pentobarbital.	*Via* GABAergic system	↑ Sleep duration	[105]
*Pinus eldarica/* n-butanolic extract	25 and 50 (mg/kg), i.p.	In mice, extracts were injected 30 min before administration of pentobarbital.	*Via* GABAergic system	↑ Sleep duration	[105]
*Stachys lavandulifolia/* extract	100 and 300 (mg/kg), i.p.	In mice, extracts were injected 30 minutes before behavioral evaluation and the administration of ketamine (100 mg/kg, i.p.).	Volatile oil and phenyl propanoid glycoside	↑ Sleep duration ↓ Locomotor activity	[112]
*Salvia leriifolia/* aqueous extract of leaf	1.15 and 1.57 (g/kg), i.p.	In mice, the effect of the extract on morphine dependence was investigated. Morphine injected sc for 3 days and on day 4 2 h before naloxone (i.p.)	GABAergic system	↑ Sleep duration	[115]
*Salvia leriifolia/* aqueous extract	0.29 (mg/kg), i.p.	In mice, the effect of the extract on morphine dependence was investigated. Morphine injected sc for 3 days and on day 4 2 h before naloxone (i.p.)	GABAergic system	↑ Muscular relaxation	[115]
*Salvia leriifolia/* ethanol extract	10, 15, and 20 (mg/kg), i.p.	In mice, evaluation of muscle relaxant was done *via* open field and traction tests	Interaction with GABAA receptor	Seductive and muscle relaxant-like effect	[270]
*Salvia reuterana*/ ethanolic extract	50, 100, and 250 (mg/kg), i.p.	In mice, extracts were injected 30 min before ketamine injection (100 mg/kg, i.p).	Inhibition of acetylcholinesterase or contact with GABA receptors	↑ Sleep duration ↓ Sleep latency	[271]
*Salvia reuterana*/ hydroalcoholic extract	100 (mg/kg), i.p.	In mice, evaluation of anxiolytic and sedative effects	Inhibition of acetylcholinesterase or contact with GABA receptors	Anxiolytic and sedative effects	[121]
*Viola tricolor/* hydroalcoholic extract	300 (mg/kg), i.p.	In mice, the extract was injected 30 min before pentobarbital (30 mg/kg, ip) injection.	Activating the GABAergic system in the basolateral amygdala and may also enhance the GABAergic systems	↑ Sleep duration no neuron toxicity	[272]
*Viola odorata*/extract	400 (mg/kg), i.p.	In rats, ketamine-induced sleep	Due to plant components	↑ Sleep duration sedative and anxiolytic effects	[147]
*Ocimum basilicum/* hydro-alcoholic extract	25, 50, or 100 (mg/kg), i.p.	In mice, all test compounds were injected 30 min before pentobarbital administration (30 mg/kg).	‎ Due to non-polar agents such as linalool, eugenol, bergamotene, germacrene D, cadinene, cadinene, selinene and spathulenol	↑ Sleep duration while sleep latency hypnotic effects	[154]
*Ocimum basilicum/* n-butanol extract	50 (mg/kg), i.p.	In mice, all test compounds were injected 30 min before pentobarbital (30 mg/kg)	‎ Non-polar agents such as linalool, eugenol, bergamotene, germacrene D, cadinene, cadinene, selinene and spathulenol	↑ Sleep duration while sleep latency hypnotic effects	[154]
Artemisia absinthium/ methanol and chloroform extracts	100, 200, and 400 (mg/kg)	-	*Via* the GABAergic system	↑ Sleep duration significantly reduced anxiety, induction time	[159]
*A. absinthium/* aqueous, ethyl acetate, and n-butanol fractions	200 (mg/kg), i.p.	In mice, extracts were injected 30 minutes before the injection of 30 mg/kg pentobarbital (i.p.).	*Via* the GABAergic system	↑ Sleep duration	[160]
*A. absinthium/* extract	100, 200 (mg/kg), i.p.	In mice, extracts were injected 30 minutes before the injection of 30 mg/kg pentobarbital (i.p.).	*Via* the GABAergic system	↑ Sleep duration	[160]
*Cucurbita pepo/* fruit extract	200 (mg/kg), i.p.	In mice, extracts were injected 30 min before sodium pentobarbital (30 mg/kg, i.p.)	*Via* the GABA receptors	↑ Sleep duration	[273]
*Lagenaria vulgaris/* fruit extract	200 (mg/kg), i.p.	In mice, extracts were injected 30 min before sodium pentobarbital (30 mg/kg, i.p.)	The GABA and opioid receptors	↑ Sleep duration	[273]
*Lagenaria vulgaris*/ Seed extract water, ethyl acetate, and n-butanol extracts	50 and 100 (mg/kg), i.p.	In mice, extracts were injected 30 min before sodium pentobarbital (30 mg/kg, i.p.)	The GABA opioid receptors	↑ Sleeping time and sleep length	[273]
*Capparis spinose* hydro-ethanolic extract	100 and 300 (mg/kg), p.o.	In rats, treatment was performed 1h before LPS administration.	By microglial activation	↓ Neuroinflammation neuroprotective impact	[274]
*Capparis spinose*/ hydro-alcoholic and n-hexane, water, and ethyl acetate extracts	30, 60, and 120 (mg/kg),	In mice, extracts were injected 30 min before pentobarbital (30 mg/kg, i.p.)	-	↑ Sleep duration	[174]
*Capparis spinose*/ dichloromethane extract	25, 50 and 100 (mg/kg), i.p.	In mice, open field and pentobarbital-induced sleep tests were used.	Through involving opioid receptors are responsible for the hypnotic effects	Sedative effects	[275]
*Brassica oleracea/* Ethyl acetate extract	50-200 (mg/kg), i.p.	In mice, 30 after administration of extract, pentobarbital (30 mg/kg, i.p.) was injected	Inhibition of oxidative stress and neuroinflammation	↑ Sleep duration ↓ Sleep latency hypnotic effect	[186]
*Portulaca oleracea*/ decoction extract	25, 50 and 75 (mg/kg), i.p.	In mice, 30 after administration of extract, pentobarbital (30 mg/kg, i.p.) was injected	Catechol isoquinolines, 2-Adrenergic receptor agonist action	↑ Sleep duration	[199]
*Portulaca oleracea*/ hydroalcoholic extract	25, 50, 75, and 100 (mg/kg), i.p.	In mice, extract was injected 30 min before pentobarbital administration (30 mg/kg)	Alkanes, sterols, and terpenoids	↑ Sleep duration ↓ Sleep latency	[200]
*Cuscuta epithymum*/ hydroalcoholic extract and its fractions (water, ethyl acetate, and n-butanol fraction)	LD_50_ value for hydroalcoholic extract: 4.8 (g/kg), i.p.	In mice, extracts were injected 30 min before pentobarbital (30 mg/kg, i.p.).	Through GABAergic System	Sedative-hypnotic effects	[204]
*Cuscuta epithymum*/ methanolic extract	2.5, 10, 25, 50 and 100 mg/kg, i.p.	In mice, evaluation of the anti-nociceptive activity of the extract	μ-opioid and GABA_A_ receptor antagonists	Anti-nociceptive activity	[276]
*Perovskia abrotanoides/* hydro-alcoholic extract	25-200 (mg/kg), i.p.	In mice, extract was injected 30 min before the administration of sodium pentobarbital (30 mg/kg)	*Via* the GABAergic system	↑ Sleep duration ↓ Sleep latency	[210]
*Perovskia abrotanoides/*n-butanol fraction	25 and 50 (mg/kg), i.p.	In mice, the extract was injected 30 min before the administration of sodium pentobarbital (30 mg/kg, i.p.)	*Via* the GABAergic system	↑ Sleep duration ↓ Sleep latency	[210]
*Tanacetum parthenium/*hydro-alcoholic extract	50-200 (mg/kg), i.p.	In mice, extracts were injected 30 min before the administration of sodium pentobarbital (30 mg/kg, i.p.)	By acting on the GABAergic system	↑ Sleep duration	[218]
*Tanacetum parthenium*/ethyl acetate extract	50 (mg/kg), i.p.	In mice, extracts were injected 30 min before the administration of sodium pentobarbital (30 mg/kg, i.p.)	By acting on the GABAergic system	↑ Sleep duration ↓ Sleep latency	[218]
*Solanum lycopersicum* hydro-alcoholic extract	25,50 and 100 (mg/kg), i.p.	In mice, the extract was injected 30 min before pentobarbital (30 mg/kg, i.p.)	Through positive allosteric regulation of the GABA_A_ receptor complex	↑ Sleep duration ↓ Sleep latency hypnotic effect	[223]
*Rosa damascene/* ethanolic and aqueous extracts	500 and 1000 mg/kg	In mice, extracts were injected 30 min before the administration of pentobarbital (30 mg/kg, i.p.)	*Via* benzodiazepine receptors	↑ Sleep duration	[277]
*Rosa damascene/* ethanol extract and its aqueous, ethyl acetate, and n-butanol fractions	250 and 500 mg/kg	In mice, extracts were injected 30 min before the administration of pentobarbital (30 mg/kg, i.p.)	*Via* benzodiazepine receptors	Hypnotic effects ↑ Sleep duration	[277]
*Crocus sativus*/ ethanolic extract	5 and 10 µg/rat	In rats, One week after MS induction by i.p. injection of EB, animals were treated with two doses of saffron extract (5 and 10 µg/rat) for a week.	Alleviated the oxidative damage	Improves learning and memory deficits and restores oxidative stress indicators	[242]
*Crocus sativus*/ ethanolic and aqueous extracts	50,100, and 200 mg/kg, i.p.	In rats, a 7-day treatment	Through the GABA(A)-benzodiazepine receptor complex	↓ Neuropathic pain	[243]
Safranal	0.025, 0.05, and 0.1 mg/kg, i.p.	In rats, a 7-day treatment	Through the GABA(A)-benzodiazepine receptor complex	↓ Neuropathic pain	[243]
*Crocus sativus*/ aqueous extract	0.56 g/kg	In mice, sleep induced by sodium pentobarbital 30 (mg/kg), i.p.	-	Anxiolytic and hypnotic effects	[246]
Safranal	0.15 and 0.35 ml/kg	In mice, sleep induced by sodium pentobarbital 30 (mg/kg), i.p.	-	Anxiolytic and hypnotic effects	[246]
*Lawsonia inermis/* ethanolic extract	0.25-2.0 g/kg	In rats, pentobarbitone-induced sleeping	By potentiating GABAergic inhibition in the CNS *via* membrane hyperpolarization, resulting in a reduction in the cortical neurons firing rate in the brain, or by directly activating GABA receptors	↑ Sleep duration	[278]
*Lawsonia inermis* flowers/ethanolic extract	500 (mg/kg), p.o.	In mice, pentobarbital-induced hypnosis	By potentiating GABAergic inhibition in the CNS *via* membrane hyperpolarization, resulting in a reduction in the cortical neurons firing rate in the brain, or by directly activating GABA receptors	Considerable muscular relaxation and reduction in the start ↑ Sleep duration	[254]
*Passiflora incarnate/* extract	A single (500 mg/kg) or repeated (250 mg/kg). p.o.	In mice, sacrifice on the second or 6^th^ day after administration	↓ mRNA expression levels of GABA receptors	↑ Immobility time, palpebral closing time, and blood melatonin levels	[263]
*Passiflora incarnate/* ethanolic extract	500 (mg/kg), p.o.	In mice, for 5 consecutive days	↑ Calretinin is released by GABAergic neurons in the hippocampus and hypothalamus, and also serum melatonin and serotonin	↑ GABAergic neuron activity and blood melatonin levels ↓ EE	[279]
*Passiflora incarnate/* extract	500 (mg/kg), i.p.	In rats	-	↑ Sleep duration ↓ Sleep latency ↓ REM	[266]

**Note:** ↓(decrease), ↑(increase), Energy expenditure (EE), ethidium bromide (EB), rapid eye movement (REM) sleep, non-rapid eye movement sleep (NREM), γ-amino butyric acid type A (GABA-A) receptors.

**Table 2:** The effect of Persian medicinal plants treatments against insomnia: Clinical trials.

Study Design	Plant	Dose/Route	Treatment	Outcomes	References
Clinical trial	*Citrus aurantium*	Inhalation	3 Consecutive Nights, 3 drops every night	Improved sleep	[42]
Randomized placebo-controlled trial	*Lactuca sativa/*seed	Capsules containing 1000 mg/ Oral	Daily treatment for 2 weeks	Improved sleep	[67]
A double-blinded randomized controlled clinical trial	*Lactuca sativa/*seed	Syrup (5 ml)/ Oral	Twice daily for 4 weeks	Improved sleep	[280]
A pilot study with a randomized, single-blind, cross-over design	*Lavandula augustifolia*/oil	Oil/ aroma	Baseline, two treatment periods and a washout period, each of 1-week duration.	Improved sleep	[281]
Clinical trial	*Lavandula augustifolia*/oil	Aroma	3 nights, each time 9 hours of aromatherapy	Improved sleep	[85]
Clinical trial	*Leonurus cardiac*/oil extract	1200 mg/day	Treatment for 28 days	Sedative effects improved anxiety and sleep disorders	[88]
Triple-blind randomized controlled clinical trial	*Citrus sinensis/*essential oil	Oral	10 drops of orange peel essential oil in a glass of water, three times a day, after each meal for 8 weeks.	Improved sleep	[100]
Two single-blind, cross-over designed observation trials	*Valeriana officinalis* (a combination of valerian and hop)	Tablets, each tablet contains 250 mg valerian extract and 60 mg hop extract	Treatment with 2 tablets in the evening.	Improved sleep	[125]
Clinical random testing	Valerian/lemon balm	The capsule contains 160 mg of the essence of *Valerian officinalis* and 80 mg of lemon balm.	Patients received two capsules daily	Improved sleep	[126]
Three-group double-blind clinical trial study	Drops of valerian	Acupressure with valerian oil 2.5%	Two drops of valerian oil for 2 minutes for three nights	Improved sleep reduce waking during the night	[127]
Clinical trial	*Viola odorata*	66 mg of *V. odorata* intranasal	Nightly before sleep for 1 month	Improved sleep	[144]
Randomized clinical trial	*Viola odorata* oil	nasal drop	-	Alter sleep start, degree of hypnotic medication, sleep quality indices, mental quality of sleep, and sleep duration	[282]
Pilot randomized double-blind placebo-controlled trial	*Viola odorata*	5 ml/syrup	Every 12 h per day for 4 weeks	↑ Sleep quality index	[283]
Observational study	*Tanacetum parthenium,* in combination with magnesium, riboflavin, and CQ10	Tablet	For 16 weeks, 1 tablet, twice a day, for the first 4 weeks, following by 12-weeks constant-dose phase of 1 tablet per day	↓ Headache occurrence and pain intensity	[217]
An experimental before and after study	*Rosa damascene/* essential oil	Inhale 5 drops of Rosa damascene essential oil	Before sleeping for 20 min for 2 weeks	↓ Sleep resistance, difficulties getting up in the morning, nightmares, and waking up throughout the night	[229]
Quasi-experimental study	*Crocus sativus/*capsule	300 mg daily	Received a daily (between 12 noon and 2 pm) intake of 300 mg saffron capsule after lunch	Improved sleep ↓ anxiety	[247]
A randomized, double-blind, placebo-controlled tria	*Crocus sativus/*extract	14 mg twice daily	14 mg twice daily for 28 days	Improved sleep	[248]
Randomized double-blind controlled study	*Crocus sativus*/extract	15.5 mg daily for six weeks	Received saffron extract (15.5 mg per day) for 6 week	Improved sleep	[284]
Placebo-controlled trial	*Ziziphus jujuba*	Capsule of *Ziziphus jujuba* (2 g daily)	Treatment for four weeks.	Improved sleep, neuroprotection, anti-oxidation activity, improving ChAT activity, ↑ ACh	[258]
A double blind randomized clinical trial	*Ziziphus jujuba*	250 mg oral jujube seed capsule	Twice a day for 21 days	Improved sleep, neuroprotection, anti-oxidation activity, improving ChAT activity, ↑ ACh	[259]
Double-blind, placebo-controlled, repeated-measures design	*Passiflora incarnate*/ flowrer	A cup of p.incarnate herbal tea	A counterbalanced order of treatments (passion flower vs placebo tea), separated by 1 week, each treatment takes one week	Improved sleep	[265]

**Note:** ↓(decrease), ↑(increase), acetylcholine (ACh), choline acetyltransferase (ChAT).

## LIST OF ABBREVIATIONS

ACS: Acute Coronary Syndrome
BZD: Benzodiazepine
CNS: Central Nervous System
GAD: Glutamate Decarboxylase
NREM: Non-rapid Eye Movement
*REM*: Rapid Eye Movement
ROS: Reactive Oxygen Species

## References

[1] Bhaskar S., Hemavathy D., Prasad S. (2016). Prevalence of chronic insomnia in adult patients and its correlation with medical comorbidities.. J. Family Med. Prim. Care.

[2] Medic G., Wille M., Hemels M. (2017). Short- and long-term health consequences of sleep disruption.. Nat. Sci. Sleep.

[3] Schwartz J., Allison M.A., Ancoli-Israel S., Hovell M.F., Patterson R.E., Natarajan L., Marshall S.J., Grant I. (2013). Sleep, type 2 diabetes, dyslipidemia, and hypertension in elderly Alzheimer’s caregivers.. Arch. Gerontol. Geriatr..

[4] Richey S.M., Krystal A.D. (2011). Pharmacological advances in the treatment of insomnia.. Curr. Pharm. Des..

[5] Smith A.J., Tett S.E. (2010). Improving the use of benzodiazepines-Is it possible? A non-systematic review of interventions tried in the last 20 years.. BMC Health Serv. Res..

[6] Lie J.D., Tu K.N., Shen D.D., Wong B.M. (2015). Pharmacological treatment of insomnia.. P&T.

[7] Krystal A.D., Prather A.A., Ashbrook L.H. (2019). The assessment and management of insomnia: An update.. World Psychiatry.

[8] Butnariu M., Quispe C., Sharifi-Rad J., Pons-Fuster E., Lopez-Jornet P., Zam W., Das T., Dey A., Kumar M., Pentea M.H., Eid A., Umbetova A., Chen J.T. (2022). Naturally-occurring bioactives in oral cancer: Preclinical and clinical studies, bottlenecks and future directions.. Front. Biosci. (Schol. Ed.).

[9] Butnariu M., Quispe C., Herrera-Bravo J., Helon P., Kukula-Koch W. (2022). Lَópez, V.; Les, F.; Vergara, C.V.; Alarcَón-Zapata, P.; Alarcَón-Zapata, B.; Martorell, M.; Pentea, M.; Dragunescu, A.A.; Samfira, I.; Yessimsiitova, Z.; Daştan, S.D.; Castillo, C.M.S.; Roberts, T.H.; Sharifi-Rad, J.; Koch, W.; Cho, W.C. The effects of thymoquinone on pancreatic cancer: Evidence from preclinical studies.. Biomed. Pharmacother..

[10] Butnariu M., Quispe C., Koirala N., Khadka S., Salgado-Castillo C.M., Akram M., Anum R., Yeskaliyeva B., Cruz-Martins N., Martorell M., Kumar M., Vasile B.R., Abdull Razis A.F., Sunusi U., Muhammad K. (2022). R.; Sharifi-Rad, J. Bioactive effects of curcumin in human immunodeficiency virus infection along with the most effective isolation techniques and type of nanoformulations.. Int. J. Nanomedicine.

[11] Butnariu M., Quispe C., Herrera-Bravo J., Sharifi-Rad J., Singh L., Aborehab N.M., Bouyahya A., Venditti A., Sen S., Acharya K., Bashiry M., Ezzat S.M., Setzer W.N., Martorell M., Mileski K.S., Bagiu I.C., Docea A.O., Calina D., Cho W.C. (2022). The Pharmacological Activities of Crocus sativus L.: A review based on the mechanisms and therapeutic opportunities of its phytoconstituents.. Oxid. Med. Cell. Longev..

[12] Hosseini A., Sahebkar A. (2017). Reversal of Doxorubicin-induced cardiotoxicity by using phytotherapy: A review.. J. Pharmacopuncture.

[13] Haghjoo E., Shojaii A., Parvizi M.M. (2019). Efficacy of topical herbal remedies for insomnia in Iranian traditional medicine. Pharmacogn Res.

[14] Moein E., Hajimehdipoor H., Hamzeloo-Moghadam M., Choopani R., Toliyat T. (2016). Review of an aloe-based formulation used in Iranian traditional medicine.. Jundishapur J. Nat. Pharm. Prod..

[15] Sánchez M., González -Burgos E., Iglesias I., Góَmez-Serranillos M.P (2020). Pharmacological update properties of Aloe vera and its major active constituents.. Molecules.

[16] Surjushe A., Vasani R., Saple D.G. (2008). Aloe vera: A short review.. Indian J. Dermatol..

[17] Ahmed S.I., Jamil S., Ismatullah H., Hussain R., Bibi S., Khandaker M.U., Naveed A., Idris A.M., Emran T.B. (2023). A comprehensive perspective of traditional Arabic or Islamic medicinal plants as an adjuvant therapy against COVID-19.. Saudi J. Biol. Sci..

[18] Jiang J.G., Huang X.J., Chen J., Lin Q.S. (2007). Comparison of the sedative and hypnotic effects of flavonoids, saponins, and polysaccharides extracted from Semen Ziziphus jujube.. Nat. Prod. Res..

[19] Kaithwas G., Dubey K., Bhtia D., Sharma A.D., Pillai K. (2007). Reversal of sodium nitrite induced impairment of spontaneous alteration by Aloe vera gel: Involvement of cholinergic system.. Pharmacologyonline.

[20] Browicz K., Zohary D. (1996). The genus Amygdalus L. (Rosaceae): Species relationships, distribution and evolution under domestication.. Genet. Resour. Crop Evol..

[21] Sfahlan A.J., Mahmoodzadeh A., Hasanzadeh A., Heidari R., Jamei R. (2009). Antioxidants and antiradicals in almond hull and shell (Amygdalus communis L.) as a function of genotype.. Food Chem..

[22] Amanzadeh Y., Hajimehdipoor H., Abedi Z., Khatamsaz M. (2016). Chemical constituents of Amygdalus spp. oil from Iran.. Res. J. Pharmacogn.

[23] Karimi Z., Firouzi M., Dadmehr M., Javad-Mousavi S.A., Bagheriani N., Sadeghpour O. (2021). Almond as a nutraceutical and therapeutic agent in Persian medicine and modern phytotherapy: A narrative review.. Phytother. Res..

[24] Mandalari G., Nueno-Palop C., Bisignano G., Wickham M.S.J., Narbad A. (2008). Potential prebiotic properties of almond (Amygdalus communis L.) seeds.. Appl. Environ. Microbiol..

[25] Kulkarni K., Kasture S.B., Mengi S.A. (2010). Efficacy study of prunus amygdalus (almond) nuts in scopolamine-induced amnesia in rats.. Indian J. Pharmacol..

[26] Gorji N., Moeini R., Memariani Z. (2018). Almond, hazelnut and walnut, three nuts for neuroprotection in Alzheimer’s disease: A neuropharmacological review of their bioactive constituents.. Pharmacol. Res..

[27] Abdollahnejad F., Mosaddegh M., Kamalinejad M., Mirnajafi-Zadeh J., Najafi F., Faizi M. (2016). Investigation of sedative and hypnotic effects of Amygdalus communis L. extract: Behavioral assessments and EEG studies on rat.. J. Nat. Med..

[28] Dalangin R., Kim A., Campbell R.E. (2020). The role of amino acids in neurotransmission and fluorescent tools for their detection.. Int. J. Mol. Sci..

[29] Ito Y., Takahashi S., Shen M., Yamaguchi K., Satoh M. (2014). Effects of L-serine ingestion on human sleep.. Springerplus.

[30] Hosseinzadeh M., Moayedi A., Chudar M.H., Rezaei K. (2019). Nutritional, anti-nutritional, and antioxidant properties of several wild Almond Species from Iran.. J. Agric. Sci. Technol..

[31] Gholamrezaie Sani L., Mohammadi M., Jalali S. (2013). J.; Abolghasemi, S.A.; Roostaie A. M., M. Extract and leaf powder effect of Artemisia annua on performance, cellular and humoral immunity in broilers.. Majallah-i Tahqiqat-i Dampizishki-i Iran.

[32] Zhai D.D., Zhong J.J. (2010). Simultaneous analysis of three bioactive compounds in Artemisia annua hairy root cultures by reversed-phase high-performance liquid chromatography-diode array detector.. Phytochem. Anal..

[33] Rai S.K., Rai K.K., Pandey-Rai S. (2021). New perspectives of the Artemisia annua bioactive compounds as an affordable cure in treatment of malaria and cancer..

[34] Perazzo F.F., Lima L.M., Maistro E.L., Carvalho J.E., Rehder V.L.G., Carvalho J.C.T. (2008). Effect of Artemisia annua L. leaves essential oil and ethanol extract on behavioral assays.. Rev. Bras. Farmacogn..

[35] Sharopov F.S., Salimov A., Numonov S., Safomuddin A., Bakri M., Salimov T., Setzer W.N., Habasi M. (2020). Chemical composition,
antioxidant, and antimicrobial activities of the essential oils from
Аrtemisia annua L. growing wild in Tajikistan.. Nat. Prod. Commun.,.

[36] Kim M.H., Lee S.M., An K.W., Lee M.J., Park D.H. (2021). Usage of natural volatile organic compounds as biological modulators of disease.. Int. J. Mol. Sci..

[37] Emadi F., Yassa N., Hadjiakhoondi A., Beyer C., Sharifzadeh M. (2011). Sedative effects of Iranian Artemisia annua in mice: Possible benzodiazepine receptors involvement.. Pharm. Biol..

[38] Jamzadfard M., Ebrahimi H. (2020). Improvement of the anxiety and
depression during using Camaneur herbal distilate: Comperhensive
survey of the antioxidant effects 3rd International Conference on
Agricultural Sciences, Medicinal Plants and Traditional Medicine
Tabilisi, Georgia,.

[39] Costa C.A.R.A., Cury T.C., Cassettari B.O., Takahira R.K., Flóَrio J.C., Costa M., Citrus aurantium L. (2013). , M. L. essential oil exhibits anxiolytic-like activity mediated by 5-HT1A-receptors and reduces cholesterol after repeated oral treatment.. BMC Complement. Altern. Med..

[40] Carvalho-Freitas M.I.R., Costa M. (2002). Anxiolytic and sedative effects of extracts and essential oil from Citrus aurantium L.. Biol. Pharm. Bull..

[41] Motaghi S., Jonaidi H., Abbasnejad M., Usofi M., Khaki M.A., Sheibani V. (2016). Behavioral and electrophysiological evidence for attenuation of CNS by aqueous extract from Citrus aurantium (CaL) flowers in rat.. Comp. Clin. Pathol..

[42] Zeighami R., Jalilolghadr S. (2014). Investigating the effect of “Citrus Aurantium” aroma on sleep quality of patients hospitalized in the coronary care unit (CCU).. Complement Med. J..

[43] Wasowski C., Marder M. (2012). Flavonoids as GABAA receptor ligands: The whole story?. J. Exp. Pharmacol..

[44] Fernandez S.P., Nguyen M., Yow T.T., Chu C., Johnston G.A.R., Hanrahan J.R., Chebib M. (2009). The flavonoid glycosides, myricitrin, gossypin and naringin exert anxiolytic action in mice.. Neurochem. Res..

[45] (2018). Önder, A. Coriander and its phytoconstituents for the beneficial effects. Potential Essent oils,.

[46] Emamghoreishi M., Khasaki M., Aazam M.F. (2005). Coriandrum sativum: evaluation of its anxiolytic effect in the elevated plus-maze.. J. Ethnopharmacol..

[47] Wei J.N., Liu Z.H., Zhao Y.P., Zhao L.L., Xue T.K., Lan Q.K. (2019). Phytochemical and bioactive profile of Coriandrum sativum L.. Food Chem..

[48] Kazempor S.F. (2015). The analgesic effects of different extracts of aerial parts of Coriandrum Sativum in mice.. Int. J. Biomed. Sci..

[49] Karami R., Hosseini M., Mohammadpour T., Ghorbani A., Sadeghnia H.R., Rakhshandeh H., Vafaee F., Esmaeilizadeh M. (2015). Effects of hydroalcoholic extract of Coriandrum sativum on oxidative damage in pentylenetetrazole-induced seizures in rats.. Iran. J. Neurol..

[50] Laribi B., Kouki K., M’Hamdi M., Bettaieb T. (2015). Coriander (Coriandrum sativum L.) and its bioactive constituents.. Fitoterapia.

[51] Emamghoreishi M., Heidari-Hamedani G. (2006). Sedative-hypnotic activity of extracts and essential oil of coriander seeds.. Iran. J. Med. Sci..

[52] Hajhashemi V., Safaei A. (2015). Hypnotic effect of Coriandrum sativum, Ziziphus jujuba, Lavandula angustifolia and Melissa officinalis extracts in mice.. Res. Pharm. Sci..

[53] Gastóَn M.S, Cid M.P, Vázquez A.M, Decarlini M.F, Demmel G.I, Rossi L.I, Aimar M.L (2016). Salvatierra, N.A. Sedative effect of central administration of Coriandrum sativum essential oil and its major component linalool in neonatal chicks.. Pharm. Biol..

[54] Hosseini M., Boskabady M.H., Khazdair M.R. (2021). Neuroprotective effects of Coriandrum sativum and its constituent, linalool: A review.. Avicenna J. Phytomed..

[55] Andrade J.C. (2021). Monteiro,ÁB.; Andrade, H.H.N.; Gonzaga, T.K.S.N.; Silva, P.R.; Alves, D.N.; Castro, R.D.; Maia, M.S.; Scotti, M.T.; Sousa, D.P.; Almeida, R.N. Involvement of GABAA receptors in the anxiolytic-like effect of hydroxycitronellal.. BioMed Res. Int..

[56] Hajlaoui H., Arraouadi S., Noumi E., Aouadi K., Adnan M., Khan M.A., Kadri A., Snoussi M. (2021). Antimicrobial, antioxidant, anti-acetylcholinesterase, antidiabetic, and pharmacokinetic properties of Carum carvi L. and Coriandrum sativum L. essential oils alone and in combination.. Molecules.

[57] Wongsamitkul N. (2017). Maldifassi, M.C.; Simeone, X.; Baur, R.; Ernst, M.; Sigel, E. α subunits in GABAA receptors are dispensable for GABA and diazepam action.. Sci. Rep..

[58] Sigel E., Ernst M. (2018). The benzodiazepine binding sites of GABAA receptors.. Trends Pharmacol. Sci..

[59] Watanabe Y., Sakurai J., Izumo N. (2019). Effect of Coriandrum Sativum L. leaf extract on the brain GABA neurons in mice.. J. Nutrit. Health Food Sci..

[60] Kim M.J., Moon Y., Tou J.C., Mou B., Waterland N.L. (2016). Nutritional value, bioactive compounds and health benefits of lettuce (Lactuca sativa L.).. J. Food Compos. Anal..

[61] Zekkori B., Khallouki F., Bentayeb A., Fiorito S., Preziuso F., Taddeo V.A., Epifano F., Genovese S. (2018). A new phytochemical and
anti-oxidant and anti-inflammatory activities of different Lactuca
sativa L. var. crispa extracts. Nat. Prod. Commun.,.

[62] Dalhat M., Amale A., Maimuna M., Bashiru I., Sirajo K. (2017). Comparative study of mineral and phytochemical analysis of soil and Lactuca sativa grown in the vicinity of cement company of Northern Nigeria (Sokoto Cement) and Usmanu Danfodiyo University Sokoto (Kwalkwalawa).. Asian J. Res. Biochem.

[63] Mampholo B.M., Maboko M.M., Soundy P., Sivakumar D. (2016). Phytochemicals and overall quality of leafy lettuce (Lactuca sativa L.) varieties grown in closed hydroponic system.. J. Food Qual..

[64] Ghorbani A., Rakhshandeh H., Sadeghnia H.R. (2013). Potentiating effects of Lactuca sativa on pentobarbital-induced sleep.. Iran. J. Pharm. Res..

[65] Kim H.D., Hong K.B., Noh D.O., Suh H.J. (2017). Sleep-inducing effect of lettuce (Lactuca sativa) varieties on pentobarbital-induced sleep.. Food Sci. Biotechnol..

[66] Kim H.W., Suh H.J., Choi H.S., Hong K.B., Jo K. (2019). Effectiveness of the sleep enhancement by green romaine lettuce (Lactuca sativa) in a Rodent Model.. Biol. Pharm. Bull..

[67] Pour Z.S., Hosseinkhani A., Asadi N., Shahraki H.R., Vafaei H., Kasraeian M., Bazrafshan K., Faraji A. (2018). Double-blind randomized placebo-controlled trial on efficacy and safety of Lactuca sativa L. seeds on pregnancy-related insomnia.. J. Ethnopharmacol..

[68] Mosavat S.H., Mirzaei H.R., Mofid B., Gharehgozlou R., Parvizi M.M., Bradley R., Pasalar M., Heydarirad G. (2022). Efficacy of lettuce seed syrup on insomnia in patients with breast cancer: A pilot double blind randomized placebo controlled clinical trial.. J. Complement. Integr. Med..

[69] Janbaz K.H., Latif M.F., Saqib F., Imran I., Zia-Ul-Haq M., De Feo V. (2013). Pharmacological Effects of Lactuca serriola L. in Experimental model of gastrointestinal, respiratory, and vascular ailments.. Evid. Based Complement. Alternat. Med..

[70] Abdul-Jalil T.Z. (2020). Lactuca serriola: Short review of its phytochemical and pharmacological profiles.. Intl. J. Drug Deliv. Technol..

[71] Awan A.F., Akhtar M.S., Anjum I., Mushtaq M.N., Fatima A., Mannan A., Ali I. (2020). Anti-oxidant and hepatoprotective effects of Lactuca serriola and its phytochemical screening by HPLC and FTIR analysis.. Pak. J. Pharm. Sci..

[72] Bouimeja B., Yetongnon K.H., Touloun O., Berrougui H., Laaradia M.A., Ouanaimi F., Chait A., Boumezzough A. (2019). Studies on antivenom activity of Lactuca serriola methanolic extract against Buthus atlantis scorpion venom by *in vivo* methods.. S. Afr. J. Bot..

[73] Ilgün S., Küpeli Akkol E., Ilhan M., içek Polat D., Baldemir Kılıç A., Coşkun M., Sobarzo-Sánchez E. (2020). اSedative effects of latexes obtained from some Lactuca L. species growing in Turkey.. Molecules.

[74] Chadha A., Florentine S. (2021). Biology, ecology, distribution and control of the invasive weed, Lactuca serriola L. (Wild Lettuce): A global review.. Plants (Basel).

[75] Manayi A., Nabavi S.M., Daglia M., Jafari S. (2016). Natural terpenoids as a promising source for modulation of GABAergic system and treatment of neurological diseases.. Pharmacol. Rep..

[76] Weston-Green K., Clunas H., Jimenez N.C. (2021). A Review of the potential use of pinene and linalool as terpene-based medicines for brain health: discovering novel therapeutics in the flavours and fragrances of cannabis.. Front. Psych..

[77] (2019). Łyczko, J.; Jałoszyński, K.; Surma, M.; Masztalerz, K.; Szumny, A. HS-SPME analysis of true lavender (Lavandula angustifolia Mill.) leaves treated by various drying methods. Molecules,.

[78] Kirimer N., Mokhtarzadeh S., Demirci B., Goger F., Khawar K.M., Demirci F. (2017). Phytochemical profiling of volatile components of Lavandula angustifolia Miller propagated under *in vitro* conditions.. Ind. Crops Prod..

[79] Cardia G.F.E., Silva-Filho S.E., Silva E.L., Uchida N.S., Cavalcante H.A.O., Cassarotti L.L., Salvadego V.E.C., Spironello R.A., Bersani-Amado C.A., Cuman R.K.N. (2018). Effect of lavender (Lavandula angustifolia) essential oil on acute inflammatory response.. Evid. Based Complement. Alternat. Med..

[80] Lَpez V., Nielsen B., Solas M., Ramيrez M.J, Jنger A.J (2017). Exploring pharmacological mechanisms of lavender (Lavandula angustifolia) essential oil on central nervous system targets.. Front. Pharmacol..

[81] Sanna M.D., Les F., Lopez V., Galeotti N. (2019). Lavender (Lavandula angustifolia Mill.) essential oil alleviates neuropathic pain in mice with spared nerve injury.. Front. Pharmacol..

[82] Alnamer R., Alaoui K., Bouidida E.H., Benjouad A., Cherrah Y. (2012). Sedative and hypnotic activities of the methanolic and aqueous extracts of Lavandula officinalis from Morocco.. Adv. Pharmacol. Sci..

[83] Nasiri L.Z., Hajimonfarednejad M., Riasatian M., Abolhassanzadeh Z., Iraji A., Vojoud M., Heydari M., Shams M. (2020). Efficacy of inhaled Lavandula Angustifolia Mill. Essential oil on sleep quality, quality of life and metabolic control in patients with diabetes mellitus type II and insomnia.. J. Ethnopharmacol..

[84] El-Saber B.G., Oluwafemi T.J., Wasef L., Shaheen H.M., Akomolafe A.P., Teibo T.K.A., Al-Kuraishy H.M., Al-Garbeeb A.I., Alexiou A., Papadakis M. (2023). A review of the bioactive components and pharmacological properties of Lavandula species.. Naunyn Schmiedebergs Arch. Pharmacol..

[85] Moeini M., Khadibi M., Bekhradi R., Mahmoudian S.A., Nazari F. (2010). Effect of aromatherapy on the quality of sleep in ischemic heart disease patients hospitalized in intensive care units of heart hospitals of the Isfahan University of Medical Sciences.. Iran. J. Nurs. Midwifery Res..

[86] Fierascu R.C., Fierascu I., Ortan A., Fierascu I.C., Anuta V., Velescu B.S., Pituru S.M., Dinu-Pirvu C.E. (2019). Leonurus cardiaca L.
as a source of bioactive compounds: An update of the European
Medicines Agency Assessment Report (2010). Biomed. Res. Int.,
2019, 2019, 4303215, eCollection.

[87] Wojtyniak K. (2013). Szymański, M.; Matławska, I. Leonurus cardiaca L. (motherwort): A review of its phytochemistry and pharmacology.. Phytother. Res..

[88] Shikov A.N., Pozharitskaya O.N., Makarov V.G., Demchenko D.V., Shikh E.V. (2011). Effect of Leonurus cardiaca oil extract in patients with arterial hypertension accompanied by anxiety and sleep disorders.. Phytother. Res..

[89] Koshovyi O., Raal A., Kireyev I., Tryshchuk N., Ilina T., Romanenko Y., Kovalenko S.M., Bunyatyan N. (2021). rtPhytochemical and psychotropic research of motherwo (Leonurus cardiaca L.) Modified Dry Extracts.. Plants.

[90] Rauwald H., Savtschenko A., Merten A., Rusch C., Appel K., Kuchta K. (2015). GABAA receptor binding assays of standardized Leonurus cardiaca and Leonurus japonicus extracts as well as their isolated constituents.. Planta Med..

[91] Hassanpouraghdam M.B., Ghorbani H., Esmaeilpour M., Alford M.H., Strzemski M., Dresler S. (2022). Diversity and distribution patterns of endemic medicinal and aromatic plants of iran: implications for conservation and habitat management.. Int. J. Environ. Res. Public Health.

[92] Ghasemian S. M.A. (2015). Phytochemistry: Antimicrobial activity and
chemical composition of essential oil of stems of Nepeta Glomerulosa
from Khorasan. Iran Seminar Org. Chem.,. https://sid.ir/paper/932273/en.

[93] Javidnia K., Miri R., Rezazadeh S.R., Soltani M., Khosravi A.R. (2008). Essential oil composition of two subspecies of Nepeta glomerulosa
Boiss. from Iran. Nat Prod Commun,.

[94] Sharma A., Cooper R., Bhardwaj G., Cannoo D.S. (2021). The genus Nepeta: Traditional uses, phytochemicals and pharmacological properties.. J. Ethnopharmacol..

[95] Hosseini A., Forouzanfar F., Rakhshandeh H. (2016). Hypnotic effect of Nepeta glomerulosa on pentobarbital-induced sleep in mice.. Jundishapur J. Nat. Pharm. Prod..

[96] Mannucci C., Calapai F., Cardia L., Inferrera G., D’Arena G., Di Pietro M., Navarra M., Gangemi S., Ventura Spagnolo E., Calapai G. (2018). Clinical pharmacology of Citrus aurantium and Citrus sinensis for the treatment of anxiety.. Evid. Based Complement. Alternat. Med..

[97] (2016). Favela-Hernández J.; Gonzáez-Santiago, O.; Ramيrez-Cabrera, M.; Esquivel-Ferriٌo, P.; Camacho-Corona, M. Chemistry and pharmacology of Citrus sinensis.. Molecules.

[98] Abdelazem R., Hefnawy H., El-Shorbagy G. (2021). Chemical composition and phytochemical screening of Citrus sinensisn (Orange) PEELS.. Zagazig J. Agricultural Res..

[99] Pimenta F.C.T.N., Neto G.C., Alves M., Pimenta M.F., Diniz J.M., de Medeiros AC., Diniz M.D. (2017). Pharmacological actions of Citrus species.. J. Citrus Pathol..

[100] Mirghafourvand M., Charandabi S.M.A., Hakimi S., Khodaie L., Galeshi M. (2016). Effect of orange peel essential oil on postpartum sleep quality: A randomized controlled clinical trial.. Eur. J. Integr. Med..

[101] (2009). Guzmán-Gutiérrez S.; Navarrete, A. Pharmacological exploration of the sedative mechanism of hesperidin identified as the active principle of Citrus sinensis flowers.. Planta Med..

[102] Moghimi A., Bolandghamat S., Iranshahi M. (2011). Effects of ethanolic extract of pine needles (Pinus eldarica Medw.) on reserpine-induced depression-like behavior in male Wistar rats.. Pharmacogn. Mag..

[103] (2014). A.M. Fallah, H.H.; Tajalizadekhoob, Y.; Mirarefin, M.; Taheri, E.; Saeednia, S.; Larijani, B.; Sharifi, F.; Fakhrzadeh, H. Determination of phenolic compounds in Pinus eldarica by HPLC.. Faslnamah-i Giyahan-i Daruyi.

[104] Iravani S., Zolfaghari B. (2014). Phytochemical analysis of Pinus eldarica bark.. Res. Pharm. Sci..

[105] Forouzanfar F., Ghorbani A., Hosseini M., Rakhshandeh H. (2016). Hydroalcoholic extract of needles of Pinus eldarica enhances pentobarbital-induced sleep: possible involvement of GABAergic system.. Avicenna J. Phytomed..

[106] Woo J., Yang H., Yoon M., Gadhe CG., Pae AN., Cho S., Lee C.J.Y.H., Yoon M., Gadhe C.G., Pae A.N., Cho S., Lee C.J. (2019). 3-Carene, a phytoncide from pine tree has a sleep-enhancing effect by targeting the GABAA-benzodiazepine receptors.. Exp. Neurobiol..

[107] Yang H. (2016). Woo, J.; Pae, A.N.; Um, M.Y.; Cho, N.C.; Park, K.D.; Yoon, M.; Kim, J.; Lee, C.J.; Cho, S. α-Pinene, a major constituent of pine tree oils, enhances non-rapid eye movement sleep in mice through GABAA-benzodiazepine receptors.. Mol. Pharmacol..

[108] Morteza-Semnani K., Akbarzadeh M., Changizi S. (2006). Essential oils composition of Stachys byzantina, S. inflata, S. lavandulifolia and S. laxa from Iran.. Flavour Fragrance J..

[109] Pirbalouti A.G., Mohammadi M. (2013). Phytochemical composition of the essential oil of different populations of Stachys lavandulifolia Vahl.. Asian Pac. J. Trop. Biomed..

[110] Delazar A., Delnavazi M.R., Nahar L., Moghadam S.B., Mojarab M., Gupta A., Williams A.S., Mukhlesur R.M., Sarker S.D., Lavandulifolioside B. (2011). A new phenylethanoid glycoside from the aerial parts of Stachys lavandulifolia Vahl.. Nat. Prod. Res..

[111] Andalib S., Vaseghi A., Vaseghi G., Naeini A.M. (2011). Sedative and hypnotic effects of Iranian traditional medicinal herbs used for treatment of insomnia.. EXCLI J..

[112] Rabbani M., Sajjadi S.E., Zarei H.R. (2003). Anxiolytic effects of Stachys lavandulifolia Vahl on the elevated plus-maze model of anxiety in mice.. J. Ethnopharmacol..

[113] Hosseinzadeh H., Sadeghnia H.R., Mohsen I., Bibi Sedigheh Fazly B. (2009). Review of the pharmacological and toxicological effects of Salvia leriifolia.. Iran. J. Basic Med. Sci..

[114] Geranmayeh J., Hashemi S.M. (2014). Contact toxicity of the essential oils from Salvia leriifolia Benth (Lamiaceae) against Lasioderma serricorne (F.).. Biharean Biol..

[115] Hosseinzadeh H.H.Z.A. (2001). Muscle relaxant and hypnotic effects of Salvia leriifolia Benth leaves extract in mice.. Iran. J. Basic Med. Sci..

[116] Luo L., Xue J., Shao Z., Zhou Z., Tang W., Liu J., Hu H., Yang F. (2023). Recent developments in Salvia miltiorrhiza polysaccharides: Isolation, purification, structural characteristics and biological activities.. Front. Pharmacol..

[117] Panahi Y., Ghanei M., Hadjiakhoondi A., Ahmadi-Koulaei S., Delnavazi M.R. (2020). Free radical scavenging principles of Salvia reuterana Boiss. Aerial Parts.. Iran. J. Pharm. Res..

[118] Amiri H., Meshkat Al Sadat M., Lari Yazdi H., Goodarzi A. (2006). Essential oil composition of Salvia reuterana Boiss.. Iran J. Med. Arom. Plants Res..

[119] Jafari E., Andalib S., Abed A., Rafieian-Kopaei M., Vaseghi G. (2015). Neuroprotective, antimicrobial, antioxidant, chemotherapeutic, and antidiabetic properties of Salvia Reuterana: A mini review.. Avicenna J. Phytomed..

[120] Vaseghi G., Andalib S., Rabbani M., Sajjadi S., Jafarian A. (2013). Hypnotic effect of Salvia reuterana Boiss for treatment of insomnia.. Faslnamah-i Giyahan-i Daruyi.

[121] Rabbani M., Sajjadi S.E., Jafarian A., Vaseghi G. (2005). Anxiolytic effects of Salvia reuterana Boiss. on the elevated plus-maze model of anxiety in mice.. J. Ethnopharmacol..

[122] Mojaverrostami S., Bojnordi M.N., Ghasemi-Kasman M., Ebrahimzadeh M.A., Hamidabadi H.G. (2018). A review of herbal therapy in multiple sclerosis.. Adv. Pharm. Bull..

[123] Ray A., Gulati K., Rehman S., Rai N., Anand R. (2021). Role of nutraceuticals as adaptogens.
In book: Nutraceuticals.

[124] Orhan I.E. (2021). A review focused on molecular mechanisms of anxiolytic effect of Valerina officinalis L. in connection with its phytochemistry through in vitro/in vivo studies.. Curr. Pharm. Des..

[125] Füssel A., Wolf A. (2000). Brattstrِm, A. Effect of a fixed valerian-Hop extract combination (Ze 91019) on sleep polygraphy in patients with non-organic insomnia: A pilot study.. Eur. J. Med. Res..

[126] Taavoni S., Nazem E.N., Haghani H. (2013). Valerian/lemon balm use for sleep disorders during menopause.. Complement. Ther. Clin. Pract..

[127] Bagheri-Nesami M., Gorji M.A.H., Rezaie S., Pouresmail Z., Cherati J.Y. (2015). Effect of acupressure with valerian oil 2.5% on the quality and quantity of sleep in patients with acute coronary syndrome in a cardiac intensive care unit.. J. Tradit. Complement. Med..

[128] Das G., Shin H.S., Tundis R., Gonçalves S., Tantengco O.A.G., Campos M.G., Acquaviva R., Malfa G.A., Romano A., Robles J.A.H., Clores M.Q., Patra J.K. (2021). plant species of sub-family Valerianaceae—A review on its effect on the central nervous system.. Plants.

[129] Sadeghnia H.R., Ghorbani Hesari T., Mortazavian S.M., Mousavi S.H., Tayarani-Najaran Z., Ghorbani A. (2014). Viola tricolor induces apoptosis in cancer cells and exhibits antiangiogenic activity on chicken chorioallantoic membrane.. BioMed Res. Int..

[130] Chandra D., Kohli G., Prasad K., Bisht G., Punetha V.D., Khetwal K.S., Devrani M.K., Pandey H.K. (2015). Phytochemical and ethnomedicinal uses of family Violaceae.. Curr. Res. Chem..

[131] Witkowska-Banaszczak E., Bylka W. (2005). Matławska, I.; Goślińska, O.; Muszyński, Z. Antimicrobial activity of Viola tricolor herb.. Fitoterapia.

[132] Mousavi S.H., Naghizade B., Pourgonabadi S., Ghorbani A. (2016). Protective effect of Viola tricolor and Viola odorata extracts on serum/glucose deprivation-induced neurotoxicity: role of reactive oxygen species.. Avicenna J. Phytomed..

[133] Piana M., Silva M.A., Trevisan G., Brum T.F., Silva C.R., Boligon A.A., Oliveira S.M., Zadra M., Hoffmeister C., Rossato M.F., Tonello R., Laporta L.V., Freitas R.B., Belke B.V., Jesus R.S., Ferreira J., Athayde M.L. (2013). Antiinflammatory effects of Viola tricolor gel in a model of sunburn in rats and the gel stability study.. J. Ethnopharmacol..

[134] Hellinger R., Koehbach J., Fedchuk H., Sauer B., Huber R., Gruber C.W., Gründemann C. (2014). Immunosuppressive activity of an aqueous Viola tricolor herbal extract.. J. Ethnopharmacol..

[135] Mortazavian S.M., Ghorbani A., Ghorbani H.T. (2012). Effect of hydro-alcoholic extracts of viola tricolor and its fractions on proliferation of cervix carcinoma cells.. IJOGI.

[136] Mortazavian S.M., Ghorbani A. (2012). Antiproliferative effect of viola tricolor on neuroblastoma cells in vitro.. Aust. J. Herb. Med..

[137] Rahimi V.B., Askari V.R., Hosseini M., Yousefsani B.S., Sadeghnia H.R. (2019). Anticonvulsant activity of viola tricolor against seizures induced by pentylenetetrazol and maximal electroshock in mice.. Iran. J. Med. Sci..

[138] Mohammadi K., Mohammadi R., Asle-Rousta M., Rahnema M., Mahmazi S. (2022). Viola tricolor hydroalcoholic extract improves behavioral deficiencies in rats exposed to chronic immobilization stress. Brazilian Arch. Biol. Tech.,.

[139] Ahvazi M., Khalighi-Sigaroodi F., Charkhchiyan M.M., Mojab F., Mozaffarian V-A., Zakeri H. (2012). Introduction of medicinal plants species with the most traditional usage in alamut region.. Iran. J. Pharm. Res..

[140] Rahimi V.B., Askari V.R., Emami S.A., Tayarani-Najaran Z. (2017). Anti-melanogenic activity of Viola odorata different extracts on B16F10 murine melanoma cells.. Iran. J. Basic Med. Sci..

[141] Habibi E., Arab-Nozari M., Elahi P., Ghasemi M., Shaki F. (2019). Modulatory effects of Viola odorata flower and leaf extracts upon oxidative stress-related damage in an experimental model of ethanol-induced hepatotoxicity.. Appl. Physiol. Nutr. Metab..

[142] Rizwan K., Khan S.A., Ahmad I., Rasool N., Ibrahim M., Zubair M., Jaafar H.Z.E., Manea R. (2019). A comprehensive review on chemical and pharmacological potential of Viola betonicifolia: A plant with multiple benefits.. Molecules.

[143] Jamshed H., Siddiqi H.S., Gilani A.H., Arslan J., Qasim M., Gul B. (2019). Studies on antioxidant, hepatoprotective, and vasculoprotective potential of Viola odorata and Wrightia tinctoria.. Phytother. Res..

[144] Feyzabadi Z., Jafari F., Kamali S.H., Ashayeri H., Badiee Aval S., Esfahani M.M., Sadeghpour O. (2014). Efficacy of Viola odorata in treatment of chronic insomnia.. Iran. Red Crescent Med. J..

[145] Mehraban M.S.A., Shirzad M., Kashani L.M.T., Ahmadian-Attari M.M., Safari A.A., Ansari N., Hatami H., Kamalinejad M. (2023). Efficacy and safety of add-on Viola odorata L. in the treatment of COVID-19: A randomized double-blind controlled trial.. J. Ethnopharmacol..

[146] Huang S., Huang Q., Zhou Z., Zhang J., Zhan Y., Liang Z. (2022). The efficacy of V. odorata extract in the treatment of insomnia: A systematic review and meta-analysis.. Front. Neurol..

[147] Monadi A., Rezaie A. (2013). Evaluation of sedative and pre-anesthetic effects of Viola odorata Linn. extract compared with diazepam in rats.. Bull Environ Pharmacol Life Sci.

[148] Feyzabadi Z., Ghorbani F., Vazani Y., Zarshenas M.M. (2017). A Critical review on phytochemistry, pharmacology of Viola odorata L. and related multipotential products in traditional persian medicine.. Phytother. Res..

[149] Ansari M., Rafiee Kh., Yasa N., Vardasbi S., Naimi S.M., Nowrouzi A. (2010). Measurement of melatonin in alcoholic and hot water extracts of Tanacetum parthenium, Tripleurospermum disciforme and Viola odorata.. Daru.

[150] Marwat S.K., Khan M.S., Ghulam S., Anwar N., Mustafa G., Usman K. (2011). Phytochemical constituents and pharmacological activities of sweet Basil-Ocimum basilicum L. (Lamiaceae).. Asian J. Chem..

[151] Ouelbani R., Bensari S., Mouas T.N., Khelifi D. (2016). Ethnobotanical investigations on plants used in folk medicine in the regions of Constantine and Mila (North-East of Algeria).. J. Ethnopharmacol..

[152] Kumar B., Bajpai V., Tiwari S., Pandey R. (2020). Phytochemistry of Plants of Genus Ocimum..

[153] Abdoly M., Farnam A., Fathiazad F., Khaki A., Khaki A.A., Ibrahimi A., Afshari F., Rastgar H. (2012). Antidepressant-like activities of Ocimum basilicum (sweet Basil) in the forced swimming test of rats exposed to electromagnetic field (EMF).. Afr. J. Pharm. Pharmacol..

[154] Askari V.R., Baradaran R.V., Ghorbani A., Rakhshandeh H. (2016). Hypnotic Effect of Ocimum basilicum on pentobarbital-induced sleep in mice.. Iran. Red Crescent Med. J..

[155] Filip S. (2014). Vidović S.; Adamović D.; Zeković Z. Fractionation of non-polar compounds of basil (Ocimum basilicum L.) by supercritical fluid extraction (SFE).. J. Supercrit. Fluids.

[156] Szopa A., Pajor J., Klin P., Rzepiela A., Elansary H.O., Al-Mana F.A., Mattar M.A., Ekiert H. (2020). Artemisia absinthium L.—Importance in the history of medicine, the latest advances in phytochemistry and therapeutical, cosmetological and culinary uses.. Plants.

[157] Obistioiu D., Cristina R.T., Schmerold I., Chizzola R., Stolze K., Nichita I., Chiurciu V. (2014). Chemical characterization by GC-MS and *in vitro* activity against Candida albicans of volatile fractions prepared from Artemisia dracunculus, Artemisia abrotanum, Artemisia absinthium and artemisia vulgaris.. Chem. Cent. J..

[158] Batiha G.E.S., Olatunde A., El-Mleeh A., Hetta H.F., Al-Rejaie S., Alghamdi S., Zahoor M., Magdy B.A., Murata T., Zaragoza-Bastida A., Rivero-Perez N. (2020). Bioactive compounds, pharmacological actions, and pharmacokinetics of wormwood (artemisia absinthium).. Antibiotics (Basel).

[159] Rezaie A., Ahmadizadeh C., Hedayat M.J., Nazeri M., Zakhireh S., Rezaie S. (2012). Study of sedation, pre-anesthetic, and anti-anxiety effects of artemisia l. extract compared with diazepam in rats.. Am. J. Sci. Res..

[160] Rakhshandeh H., Heidari A., Pourbagher-Shahri A.M., Rashidi R., Forouzanfar F. (2021). Hypnotic effect of A. Absinthium hydroalcoholic extract in pentobarbital-tTreated mice.. Neurol. Res. Int..

[161] Ur Rashid M., Alamzeb M., Ali S., Ullah Z., Shah Z.A., Naz I., Khan M.R. (2019). The chemistry and pharmacology of alkaloids and allied nitrogen compounds from Artemisia species: A review.. Phytother. Res..

[162] Prajapati R., Kalariya M., Parmar S., Sheth N. (2010). Phytochemical and pharmacological review of Lagenaria sicereria.. J. Ayurveda Integr. Med..

[163] Mukherjee P.K., Singha S., Kar A., Chanda J., Banerjee S., Dasgupta B., Haldar P.K., Sharma N. (2022). Therapeutic importance of Cucurbitaceae: A medicinally important family.. J. Ethnopharmacol..

[164] Haghjoo E., Haghighi K.S., Dabaghian F.H., Shojaii A., Mohammadi H. (2019). Efficacy of pumpkin oil (a Persian medicine product) in the treatment of chronic insomnia: A randomized double-blind clinical trial.. J. Pharm. Pharmacogn. Res..

[165] Rajasree R., Sibi P., Francis F., William H. (2016). Phytochemicals of Cucurbitaceae family—A review.. Int. J. Pharmacogn. Phytochem. Res..

[166] Prajapati R.P., Kalaria M.V., Karkare V.P., Parmar S.K., Sheth N.R. (2011). Effect of methanolic extract of Lagenaria siceraria (Molina) Standley fruits on marble-burying behavior in mice: Implications for obsessive-compulsive disorder.. Pharmacognosy Res..

[167] Mayakrishnan V., Veluswamy S., Sundaram K.S., Kannappan P., Abdullah N. (2013). Free radical scavenging potential of Lagenaria siceraria (Molina) Standl fruits extract.. Asian Pac. J. Trop. Med..

[168] Antoniou V., Gauhar V., Modi S., Somani B.K. (2023). Role of Phytotherapy in the Management of BPH: A Summary of the Literature.. J. Clin. Med..

[169] Gazola A.C., Costa G.M., Castellanos L., Ramos F.A., Reginatto F.H., Lima T.C.M., Schenkel E.P. (2015). Involvement of GABAergic pathway in the sedative activity of apigenin, the main flavonoid from Passiflora quadrangularis pericarp.. Rev. Bras. Farmacogn..

[170] Chedraoui S., Abi-Rizk A., El-Beyrouthy M., Chalak L., Ouaini N., Rajjou L. (2017). Capparis spinosa L. in a systematic review: A Xerophilous species of multi values and promising potentialities for agrosystems under the threat of global warming.. Front. Plant Sci..

[171] Zhang H., Ma Z. (2018). Phytochemical and pharmacological properties of Capparis spinosa as a medicinal plant.. Nutrients.

[172] Tir M., Feriani A., Labidi A., Mufti A., Saadaoui E., Nasri N., Khaldi A., El Cafsi M., Tlili N. (2019). Protective effects of phytochemicals of Capparis spinosa seeds with cisplatin and CCl4 toxicity in mice.. Food Biosci..

[173] Olas B. (2023). The current state of knowledge about the biological activity of different parts of capers.. Nutrients.

[174] Rakhshandeh H., Rashidi R., Vahedi M.M., Khorrami M.B., Abbassian H., Forouzanfar F. (2021). Hypnotic activity of Capparis spinosa hydro-alcoholic extract in mice.. Recent Pat. Food Nutr. Agric..

[175] Faizi M. (2021). Sedative-hypnotic effects of different extracts and fractions
of Capparis Spinosa L. in Mice. Intl. Pharmacy Acta,.

[176] Draghici G., Alexandra L.M., Aurica–Breica B., Nica D., Alda S., Liana A., Gogoasa I., Gergen I., Despina-Maria B. (2013). Red cabbage, millennium’s functional food.. J. Hortic. For. Biotechnol..

[177] Ahmed M.F., Rao A.S., Ahemad S.R., Ibrahim M. (2012). Phytochemical studies and antioxidant activities of Brassica oleracea L. Var.. Capitata. Int. J. Pharm. Pharm. Sci..

[178] Talreja K., Moon A. (2014). Brassica oleracea: phytochemical profiling in Search for anticancer compound. Int. J. Sci. Pharm. Res.,.

[179] Jana S., Patel D., Patel S., Upadhyay K., Thadani J., Mandal R., Das S., Devkar R. (2017). Anthocyanin rich extract of Brassica oleracea L. alleviates experimentally induced myocardial infarction.. PLoS One.

[180] Kataya H.A.H., Hamza A.A. (2008). Red cabbage (Brassica oleracea) ameliorates diabetic nephropathy in rats.. Evid. Based Complement. Alternat. Med..

[181] Sankhari J.M., Thounaojam M.C., Jadeja R.N., Devkar R.V., Ramachandran A.V. (2012). Anthocyanin-rich red cabbage (Brassica oleracea L.) extract attenuates cardiac and hepatic oxidative stress in rats fed an atherogenic diet.. J. Sci. Food Agric..

[182] Veber B., Camargo A., Dalmagro A.P., Bonde H.L.P., Magro D.D.D., Lima D.D.D., Zeni A.L.B. (2020). Red cabbage (Brassica oleracea L.) extract reverses lipid oxidative stress in rats.. An. Acad. Bras. Cienc..

[183] Zhang N., Jiao S., Jing P. (2021). Red cabbage rather than green cabbage increases stress resistance and extends the lifespan of Caenorhabditis elegans.. Antioxidants.

[184] Masci A., Mattioli R., Costantino P., Baima S., Morelli G., Punzi P., Giordano C., Pinto A., Donini L.M., d’Erme M., Mosca L. (2015). Neuroprotective effect of Brassica oleracea sprouts crude juice in a cellular model of Alzheimer’s Disease.. Oxid. Med. Cell. Longev..

[185] Nam M.K., Kang K.J. (2013). The effect of red cabbage (Brassica oleracea L. var. capitata f. rubra) extract on the apoptosis in human breast cancer MDA-MB-231 cells. J. Korean Soc.. Food Sci. Nutrition.

[186] Rakhshandeh H., Hosseini A., Sobhanifar M-A., Forouzanfar F., Aghaee A. (2018). Hypnotic effect of red cabbage (Brassica oleracea) on pentobarbital-induced sleep in mice.. J. Pharm. Bioallied Sci..

[187] Ortega-Hernández E., Antunes-Ricardo M., Jacobo-Velázquez D.A. (2021). Improving the health-benefits of kales (Brassica oleracea L. var. acephala DC) through the application of controlled abiotic stresses: A Review.. Plants.

[188] Zhou Y.X., Xin H.L., Rahman K., Wang S.J., Peng C., Zhang H. (2015). Portulaca oleracea L.: A review of phytochemistry and pharmacological effects.. BioMed Res. Int..

[189] Jelodar G.A., Boskabady M.H., Yahyazadeh M. (2018). S.N.; Askari, V.R.; Ghorani, V. The effect of Portulaca oleracea and α-linolenic acid on oxidant/antioxidant biomarkers of human peripheral blood mononuclear cells.. Indian J. Pharmacol..

[190] Rahimi V.B., Ajam F., Rakhshandeh H., Askari V.R. (2019). A Pharmacological Review on Portulaca oleracea L.: Focusing on anti-inflammatory, anti- oxidant, immuno-modulatory and antitumor activities.. J. Pharmacopuncture.

[191] Jaafari A., Baradaran R.V., Vahdati-Mashhadian N., Yahyazadeh R., Ebrahimzadeh-Bideskan A., Hasanpour M., Iranshahi M., Ehtiati S., Rajabi H., Mahdinezhad M., Rakhshandeh H., Askari V.R. (2021). Evaluation of the therapeutic effects of the hydroethanolic extract of portulaca oleracea on surgical-induced peritoneal adhesion.. Mediators Inflamm..

[192] Hashemzehi M., Khazdair M.R., Kiyanmehr M., Askari V.R., Boskabady M.H. (2016). Portulaca oleracea affects muscarinic receptors of Guinea pig tracheal smooth muscle.. Indian J. Pharm. Sci..

[193] Boskabady M.H., Hashemzehi M., Khazdair M.R., Askari V.R. (2016). Hydro-ethanolic extract of Portulaca oleracea affects beta-adrenoceptors of guinea pig tracheal smooth muscle.. Iran. J. Pharm. Res..

[194] Baradaran Rahimi V., Rakhshandeh H., Raucci F., Buono B., Shirazinia R., Samzadeh Kermani A., Maione F., Mascolo N., Askari V.R. (2019). Anti-inflammatory and anti-oxidant activity of Portulaca oleracea extract on lps-induced rat lung injury.. Molecules.

[195] Baradaran Rahimi V., Mousavi S.H., Haghighi S., Soheili-Far S., Askari V.R. (2019). Cytotoxicity and apoptogenic properties of the standardized extract of Portulaca oleracea on glioblastoma multiforme cancer cell line (U-87): A mechanistic study.. EXCLI J..

[196] Baradaran R.V., Askari V.R. (2021). Promising anti-melanogenic impacts of Portulaca oleracea on B16F1 murine melanoma cell line: An in-vitro vision.. S. Afr. J. Bot..

[197] Butnariu M. (2018). Portulaca Oleracea phytochemistry and pharmacological considerations.. Ann. Pharmacol. Pharm..

[198] Abdel Moneim A.E., Dkhil M.A., Al-Quraishy S. (2013). The potential role of Portulaca oleracea as a neuroprotective agent in rotenone-induced neurotoxicity and apoptosis in the brain of rats.. Pestic. Biochem. Physiol..

[199] Miladi-Gorji H., Vafaei A.A., Bageri A. (2011). To investigate the effect of Portulaca oleracea L. and Melissa officinalis L. extract on sleeping time in mice.. Faslnamah-i Giyahan-i Daruyi.

[200] Hamedi S., Forouzanfar F., Rakhshandeh H., Arian A. (2019). Hypnotic Effect of Portulaca oleracea on pentobarbital-induced sleep in mice.. Curr. Drug Discov. Technol..

[201] Kumar A., Sreedharan S., Kashyap A.K., Singh P., Ramchiary N. (2022). A review on bioactive phytochemicals and ethnopharmacological potential of purslane (Portulaca oleracea L.).. Heliyon.

[202] Olszewski M. (2019). Diversity and evolution of seeds in Cuscuta (dodders,
Convolvulaceae): Morphology and structure. Thesis. Biology,.

[203] Chabra A., Monadi T., Azadbakht M., Haerizadeh S.I. (2019). Ethnopharmacology of Cuscuta epithymum: A comprehensive review on ethnobotany, phytochemistry, pharmacology and toxicity.. J. Ethnopharmacol..

[204] Forouzanfar F., Vahedi M.M., Aghaei A., Rakhshandeh H. (2020). Hydroalcoholic extract of Cuscuta Epithymum enhances pentobarbitalinduced sleep: Possible involvement of GABAergic system.. Curr. Drug Discov. Technol..

[205] Ahmad A., Tandon S., Xuan T.D., Nooreen Z. (2017). A review on phytoconstituents and biological activities of Cuscuta species.. Biomed. Pharmacother..

[206] Ghafourian M., Mazandarani M. (2016). Ethnopharmacology, ecological requirements, antioxidant and antimicrobial activities of Perovskia abrotanoides Karel. extract for vaginal infections from semnan province.. Int. J. Women’s Health Reprod. Sci..

[207] Ghaderi S., Nejad Ebrahimi S., Ahadi H., Eslambolchi M.S., Mirjalili M.H. (2019). In vitro propagation and phytochemical assessment of Perovskia abrotanoides Karel. (Lamiaceae) – A medicinally important source of phenolic compounds.. Biocatal. Agric. Biotechnol..

[208] Beikmohammadi M. (2012). The evaluation of medicinal properties of Perovskia abrotanoides Karel.. Middle East J. Sci. Res..

[209] Khaliq S., Volk F.J., Frahm A. (2007). Phytochemical investigation of Perovskia abrotanoides.. Planta Med..

[210] Forouzanfar F., Hosseini A., Amiri M.S., Rakhshandeh H. (2017). Potentiating effects of Perovskia abrotanoides Karel. on pentobarbital-induced sleep.. Avicenna J. Phytomed..

[211] Doyno C.R., White C.M. (2021). Sedative-hypnotic agents that impact Gamma-aminobutyric acid receptors: focus on Flunitrazepam, Gamma-hydroxybutyric acid, phenibut, and selank.. J. Clin. Pharmacol..

[212] Rezaei F., Jamei R., Heidari R. (2017). Evaluation of the phytochemical and antioxidant potential of aerial parts of Iranian Tanacetum parthenium.. Ulum-i Daruyi.

[213] Hekmat Sorush I., Milani Kalkhorani N., Rezaee M. B., Hero Abadi F., Hamisi M. (2014). Phytochemical analysis of essential oil of
Tanacetum parthenium L. With hydro-distillation and steam distillation.
J. Med. Plant. By-product,.

[214] Williams C.A., Hoult J.R.S., Harborne J.B., Greenham J., Eagles J. (1995). A biologically active lipophilic flavonol from Tanacetum parthenium.. Phytochemistry.

[215] Rateb ME.-G.A., El-Hawary S. (2008). El - Shamy A. Phytochemical and biological studies on the different organs of Tanacetum parthenium L. cultivated in Egypt.. Faslnamah-i Giyahan-i Daruyi.

[216] Pareek A., Suthar M., Rathore G., Bansal V. (2011). Fever few (Tanacetum parthenium L.): A systematic review.. Pharmacogn. Rev..

[217] Moscano F., Guiducci M., Maltoni L., Striano P., Ledda M.G., Zoroddu F., Raucci U., Villa M.P., Parisi P. (2019). An observational study of fixed-dose Tanacetum parthenium nutraceutical preparation for prophylaxis of pediatric headache.. Ital. J. Pediatr..

[218] Forouzanfar F., Ghazavi H., Vahedi M.M., Tarrah K., Yavari Z., Hosseini A., Aghaee A., Rakhshandeh H. (2020). Tanacetum parthenium enhances pentobarbital-induced sleeping behaviors.. Avicenna J. Phytomed..

[219] Cárdenas J., Reyes-Pérez V., Hernández-Navarro M.D, Dorantes-Barrَn A.M, Almazán S., Estrada-Reyes R. (2017). Anxiolytic- and antidepressant-like effects of an aqueous extract of Tanacetum parthenium L. Schultz-Bip (Asteraceae) in mice.. J. Ethnopharmacol..

[220] (2020). Martínez, J.P.; Fuentes, R.; Farيas, K.; Lizana, C.; Alfaro, J.F.; Fuentes, L.; Calabrese, N.; Bigot, S.; Quinet, M.; Lutts, S. Effects of salt stress on fruit antioxidant capacity of wild (Solanum chilense) and domesticated (Solanum lycopersicum var. cerasiforme) tomatoes.. Agronomy (Basel).

[221] Ferrer-Dubois A.E., Fung-Boix Y. (2018). Isaac-Alemán, E.; Beenaerts, N.; Cuypers, A. Phytochemical determination of Solanum lycopersicum L. fruits irrigated with water treated with static magnetic field. Rev. Cuba.. Quيm..

[222] Hussain G., Rasul A., Anwar H., Aziz N., Razzaq A., Wei W., Ali M., Li J., Li X. (2018). Role of plant derived alkaloids and their mechanism in neurodegenerative disorders.. Int. J. Biol. Sci..

[223] Molkara T., Forouzanfar F., Hamedi S., Aghaee A., Goldoozian R., Rakhshandeh H. (2018). Hypnotic effect of solanum lycopersicumand solanum nigrumon pentobarbital-induced sleep in mice.. Plant Arch..

[224] Nonaka S., Arai C., Takayama M., Matsukura C., Ezura H. (2017). Efficient increase of ɣ-aminobutyric acid (GABA) content in tomato fruits by targeted mutagenesis.. Sci. Rep..

[225] Ansari S., Zeenat F., Ahmad W., Ahmad I. (2017). Therapeutics and pharmacology of Gul-e-Surkh (Rosa damascena Mill): An important Unani drug.. Int. J. Adv. Pharm. Med. Bioallied Sci..

[226] Piotrowicz Z. (2021). Tabisz, Ł.; Waligََórska, M.; Pankiewicz, R.; Łęska, B. Phenol-rich alternatives for Rosa x damascena Mill. Efficient phytochemical profiling using different extraction methods and colorimetric assays.. Sci. Rep..

[227] Kamali M., Seifadini R., Kamali H., Mehrabani M., Jahani Y., Tajadini H. (2018). Efficacy of combination of Viola odorata, Rosa damascena and Coriandrum sativum in prevention of migraine attacks: A randomized, double blind, placebo-controlled clinical trial.. Electron. Physician.

[228] Ankarali S., Beyazcicek E., Kilinc E., Beyazcicek O., Ozkan K., Cetinkaya A., Cangur S., Ankarali H. (2018). The effect of rose oil on penicillin-induced epileptiform activity in rats: An electrophysiological study.. Konuralp Tip Derg..

[229] Keyhanmehr A.S., Movahhed M., Sahranavard S., Gachkar L., Hamdieh M., Afsharpaiman S., Nikfarjad H. (2018). The effect of aromatherapy with Rosa damascena essential oil on sleep quality in children.. Res. J. Pharmacogn..

[230] Sanatkaran A., Bahari F., Ansari A., Atashi N. (2016). The effect of red rose essential oil and lavender aromatherapy on the frequency of lucid dreaming, recalling dreams and sleep quality in female students. Mediterranean J. Social Sci..

[231] Ghorbani Rami M.S., Nasiri M., Aghili Nasab M.S., Jafari Z., Torkaman M., Feizi S., Farahmandnia B., Asadi M. (2021). Effect of Rosa damascena on improvement of adults’ sleep quality: A systematic review and meta-analysis of randomized controlled trials.. Sleep Med..

[232] Mohamadi N., Pourkorrani M.H.S., Langarizadeh M.A., Ranjbartavakoli M., Sharififar F., Asgary S. (2022). Evidence for Rosa damascena efficacy in mental disorders in preclinical animal studies and clinical trials: A systematic review.. Phytother. Res..

[233] Rakhshandah H., Shakeri M.T., Ghasemzadeh M.R. (2010). Comparative hypnotic effect of Rosa damascena fractions and Diazepam in Mice.. Iran. J. Pharm. Res..

[234] Boskabady M.H., Shafei M.N., Saberi Z., Amini S. (2011). Pharmacological effects of Rosa damascena.. Iran. J. Basic Med. Sci..

[235] Ulbricht C., Conquer J., Costa D., Hollands W., Iannuzzi C., Isaac R., Jordan J.K., Ledesma N., Ostroff C., Serrano J.M.G., Shaffer M.D., Varghese M. (2011). An evidence-based systematic review of saffron (Crocus sativus) by the Natural Standard Research Collaboration.. J. Diet. Suppl..

[236] Rahmanian-Devin P., Rakhshandeh H., Baradaran R.V., Sanei-Far Z., Hasanpour M., Memarzia A., Iranshahi M., Askari V.R. (2021). Intraperitoneal lavage with Crocus sativus prevents postoperative-induced peritoneal adhesion in a rat model: evidence from animal and cellular studies.. Oxid. Med. Cell. Longev..

[237] Baradaran Rahim V., Khammar M.T., Rakhshandeh H., Samzadeh-Kermani A., Hosseini A., Askari V.R. (2019). Crocin protects cardiomyocytes against LPS-induced inflammation.. Pharmacol. Rep..

[238] Mollazadeh H., Emami S.A., Hosseinzadeh H. (2015). Razi’s Al-Hawi and saffron (Crocus sativus): A review.. Iran. J. Basic Med. Sci..

[239] Rouhi B.H., Kiani S. (2016). Therapeutic effects of Crocus sativus: An overview of systematic reviews.. Future Nat Prod.

[240] Hatziagapiou K., Lambrou G.I. (2020). Anti-toxicant properties of saffron and relevance to protection from toxins and drugs.. Curr. Bioact. Compd..

[241] Bian Y., Zhao C., Lee S.M.Y. (2020). Neuroprotective potency of saffron against neuropsychiatric diseases, neurodegenerative diseases, and other brain disorders: From bench to bedside.. Front. Pharmacol..

[242] Ghaffari Sh., Hatami H., Dehghan G. (2015). Saffron ethanolic extract attenuates oxidative stress, spatial learning, and memory impairments induced by local injection of ethidium bromide.. Res. Pharm. Sci..

[243] Amin B., Hosseinzadeh H. (2012). Evaluation of aqueous and ethanolic extracts of saffron, Crocus sativus L., and its constituents, safranal and crocin in allodynia and hyperalgesia induced by chronic constriction injury model of neuropathic pain in rats.. Fitoterapia.

[244] Mohajeri S.A., Sepahi S., Azam A.G. (2020). Chapter 27 - Antidepressant and antianxiety properties of saffron..

[245] Hosseinzadeh H., Sadeghnia H.R. (2007). Protective effect of safranal on pentylenetetrazol-induced seizures in the rat: Involvement of GABAergic and opioids systems.. Phytomedicine.

[246] Hosseinzadeh H., Noraei N.B. (2009). Anxiolytic and hypnotic effect of Crocus sativus aqueous extract and its constituents, crocin and safranal, in mice.. Phytother. Res..

[247] Shahdadi H., Balouchi A., Dehghanmehr S. (2018). Effect of saffron oral capsule on anxiety and quality of sleep of diabetic patients in a tertiary healthcare facility in Southeastern Iran: A quasi-experimental study.. Trop. J. Pharm. Res..

[248] Lopresti A.L., Smith S.J., Metse A.P., Drummond P.D. (2020). Effects of saffron on sleep quality in healthy adults with self-reported poor sleep: A randomized, double-blind, placebo-controlled trial.. J. Clin. Sleep Med..

[249] (2022). Cerdá-Bernad D.; Costa, L.; Serra, A.T.; Bronze, M.R.; Valero-Cases, E.; Pérez-Llamas, F.; Candela, M.E.; Arnao, M.B.; Barberán, F.T.; Villalba, R.G.; Garcيa-Conesa, M-.T.; Frutos, M-.J. Saffron against neuro-cognitive disorders: an overview of its main bioactive compounds, their metabolic fate and potential mechanisms of neurological protection.. Nutrients.

[250] Al-Snafi A.E. (2019). A review on Lawsonia inermis: A potential medicinal plant.. Int. J. Curr. Pharm. Res..

[251] Audu B., Yusuf S., Malachy N., Jamiu O., Wade J. (2018). Phytochemical, proximate and sedative properties of henna (Lawsonia inermis) on the opercula ventilation rate of Tilapia zilli fingerlings.. Sci. World J..

[252] Rakhshandeh H., Ghorbanzadeh A., Negah S.S., Akaberi M., Rashidi R., Forouzanfar F. (2021). Pain-relieving effects of Lawsonia inermis on neuropathic pain induced by chronic constriction injury.. Metab. Brain Dis..

[253] Akram M., Hamid A., Khalil A., Ghaffar A., Tayyaba N., Saeed A., Ali M., Naveed A. (2014). Review on medicinal uses, pharmacological, phytochemistry and immunomodulatory activity of plants.. Int. J. Immunopathol. Pharmacol..

[254] Syeda N.F., Hemalatha G., Smitha T., Rama M. (2020). Assessment of neuropharmacological profile of ethanolic extract of Lawsonia Inermis flowers.. Mapana J Sci.

[255] Nesa L., Munira S., Mollika S., Islam M., Choin H., Chouduri A.U., Naher N. (2014). Evaluation of analgesic, anti-inflammatory and CNS depressant activities of methanolic extract of Lawsonia inermis barks in mice.. Avicenna J. Phytomed..

[256] Rashwan A.K., Karim N., Shishir M.R.I., Bao T., Lu Y., Chen W. (2020). Jujube fruit: A potential nutritious fruit for the development of functional food products.. J. Funct. Foods.

[257] Chen J., Liu X., Li Z., Qi A., Yao P., Zhou Z., Dong T.T.X., Tsim K.W.K. (2017). A Review of Dietary Ziziphus jujuba Fruit (Jujube): developing health food supplements for brain protection.. Evid. Based Complement. Alternat. Med..

[258] Shergis J.L., Hyde A., Meaklim H., Varma P., Da Costa C., Jackson M.L. (2021). Medicinal seeds Ziziphus spinosa for insomnia: A randomized, placebo-controlled, cross-over, feasibility clinical trial.. Complement. Ther. Med..

[259] Mahmoudi R., Ansari S., Haghighizadeh M.H., Shakiba M.N., Montazeri S. (2020). Investigation the effect of jujube seed capsule on sleep quality of postmenopausal women: A double-blind randomized clinical trial.. Biomedicine (Taipei).

[260] Yang B., Yang H., Chen F., Hua Y., Jiang Y. (2013). Phytochemical analyses of Ziziphus jujuba Mill. var. spinosa seed by ultrahigh performance liquid chromatography-tandem mass spectrometry and gas chromatography-mass spectrometry.. Analyst (Lond.).

[261] Patel S., Verma N., Gauthaman K. (2009). Passiflora incarnata Linn: A review on morphology, phytochemistry and pharmacological aspects.. Pharmacogn. Rev..

[262] Smruthi R.D.M., Archana K., Ravi M. (2021). The active compounds of Passiflora spp and their potential medicinal uses from both *in vitro* and *in vivo* evidences.. J. Pharm. Pharm. Sci..

[263] Kim G.H., Kim Y., Yoon S., Kim S.J., Yi S.S. (2020). Sleep-inducing effect of Passiflora incarnata L. extract by single and repeated oral administration in rodent animals.. Food Sci. Nutr..

[264] Pereira Z.C., Cruz J.M.D.A., Corrêa R.F., Sanches E.A., Campelo P.H., Bezerra J.d.A. (2023). Passion fruit (Passiflora spp.) pulp: A review on bioactive properties, health benefits and technological potential.. Food Res. Int..

[265] Ngan A., Conduit R. (2011). A double-blind, placebo-controlled investigation of the effects of Passiflora incarnata (passionflower) herbal tea on subjective sleep quality.. Phytother. Res..

[266] Guerrero F.A., Medina G.M. (2017). Effect of a medicinal plant (Passiflora incarnata L) on sleep.. Sleep Sci..

[267] Welz A.N., Emberger-Klein A., Menrad K. (2018). Why people use herbal medicine: insights from a focus-group study in Germany.. BMC Complement. Altern. Med..

[268] Abdollahnejad F., Mosaddegh M., Nasoohi S., Mirnajafi-Zadeh J., Kamalinejad M., Faizi M. (2016). Study of sedative-hypnotic effects of Aloe vera L. Aqueous extract through behavioral evaluations and eeg recording in rats.. Iran. J. Pharm. Res..

[269] Hosseini A., Ghorbani A., Sadeghnia H.R., Rajabian A., Rakhshandeh H. (2014). Potentiating effects of Lactuca serriola on pentobarbital-induced sleep.. Res. Opin. Anim. Vet. Sci..

[270] Hussain A., Rauf A., Abu-Izneid T., Ibrahim M., Abrar S., Khan H., Barkath Ullah, Cerón-Carrasco J.P., Pérez-Sánchez H., Choudhary M.I, Mubarak M.S, Shariati M.A, Mabkhot N.Y, Bourguet-Kondracki M.L (2020). Sedative, muscle relaxant-like effects, and molecular docking study of compounds isolated from Salvia leriifolia.. Rev. Bras. Farmacogn..

[271] Vaseghi G., Andalib S., Rabbani M., Sajjadi S., Jafarian A. (2013). Hypnotic effect of Salvia reuterana Boiss for treatment of insomnia.. J. Med. Plants.

[272] Ahmad G., Naimeh J.Y., Hassan R. (2012). Effect of Viola tricolor on pentobarbital-induced sleep in mice.. Afr. J. Pharm. Pharmacol..

[273] Baradaran Rahimi V., Askari V., Tajani A., Hosseini A., Rakhshandeh H. (2018). Evaluation of the sleep-prolonging effect of Lagenaria vulgaris and Cucurbita pepo extracts on pentobarbital-induced sleep and possible mechanisms of action.. Medicina (Kaunas).

[274] Baradaran R.V., Rajabian A., Rajabi H., Mohammadi Vosough E., Mirkarimi H.R., Hasanpour M., Iranshahi M., Rakhshandeh H., Askari V.R. (2020). The effects of hydro-ethanolic extract of Capparis spinosa (C. spinosa) on lipopolysaccharide (LPS)-induced inflammation and cognitive impairment: Evidence from *in vivo* and *in vitro* studies.. J. Ethnopharmacol..

[275] Khoramjouy M. (2021). Sedative-hypnotic effects of different extracts and fractions of Capparis spinosa L. in mice.. Int Pharm Acta.

[276] Khakpour T.B., Ghaderi B., Rostampour M., Fekjur E.M., Hasannejad F., Ansar M.M., 1232 Current Neuropharmacology, 2024, Vol. 22, No. 7 Hosseini et al. (2021). Involvement of opioidergic and GABAergic systems in the anti-nociceptive activity of the methanolic extract of Cuscuta epithymum Murr. in mice.. J. Ethnopharmacol..

[277] Rakhshandah H., Hosseini M., Doulati K. (2004). Hypnotic effect of Rosa damascena in mice.. Iran. J. Pharm. Res..

[278] Alia B.H., Bashir A.K., Tanira M.O.M. (1995). Anti-inflammatory, antipyretic, and analgesic effects of Lawsonia inermis L. (henna) in rats.. Pharmacology.

[279] Kim G.H., Yi S.S. (2019). Chronic oral administration of Passiflora incarnata extract has no abnormal effects on metabolic and behavioral parameters in mice, except to induce sleep.. Lab. Anim. Res..

[280] Shi M., Gu J., Wu H., Rauf A., Emran T.B., Khan Z., Mitra S., Aljohani A.S.M., Alhumaydhi F.A., Al-Awthan Y.S., Bahattab O., Thiruvengadam M., Suleria H.A.R. (2022). Phytochemicals, nutrition, metabolism, bioavailability, and health benefits in lettuce-A comprehensive review.. Antioxidants (Basel).

[281] Lewith G.T., Godfrey A.D., Prescott P. (2005). A single-blinded, randomized pilot study evaluating the aroma of Lavandula augustifolia as a treatment for mild insomnia.. J. Altern. Complement. Med..

[282] Hejazian M.S., Ganjloo J., Ghorat F., Rastaghi S. (2018). Effect of Viola odorata nasal drop on sleep quality of older adults.. J. Res. Med. Dent. Sci..

[283] Shayesteh M., Vaez-Mahdavi M.R., Shams J., Kamalinejad M., Faghihzadeh S., Gholami-Fesharaki M., Gharebaghi R., Heidary F. (2020). Effects of Viola odorata as an add-on therapy on insomnia in patients with obsession or depression: A pilot randomized double-blind placebo-controlled trial.. J. Altern. Complement. Med..

[284] Pachikian B.D., Copine S., Suchareau M., Deldicque L. (2021). Effects of saffron extract on sleep quality: A randomized double-blind controlled clinical trial.. Nutrients.

